# Effect of Low-Input Organic and Conventional Farming Systems on Maize Rhizosphere in Two Portuguese Open-Pollinated Varieties (OPV), “Pigarro” (Improved Landrace) and “SinPre” (a Composite Cross Population)

**DOI:** 10.3389/fmicb.2021.636009

**Published:** 2021-02-26

**Authors:** Aitana Ares, Joana Costa, Carolina Joaquim, Duarte Pintado, Daniela Santos, Monika M. Messmer, Pedro M. Mendes-Moreira

**Affiliations:** ^1^Department of Life Sciences, Centre for Functional Ecology, University of Coimbra, Coimbra, Portugal; ^2^Laboratory for Phytopathology, Instituto Pedro Nunes, Coimbra, Portugal; ^3^Centro de Recursos Naturais, Ambiente e Sociedade (CERNAS), Coimbra, Portugal; ^4^Research Institute of Organic Agriculture (FiBL), Frick, Switzerland; ^5^Instituto Politécnico de Coimbra, Escola Superior Agrária de Coimbra, Coimbra, Portugal

**Keywords:** maize, microbiota, rhizosphere, organic and conventional farming system, open-pollinated populations, next-generation sequencing

## Abstract

Maize is one of the most important crops worldwide and is the number one arable crop in Portugal. A transition from the conventional farming system to organic agriculture requires optimization of cultivars and management, the interaction of plant–soil rhizosphere microbiota being pivotal. The objectives of this study were to unravel the effect of population genotype and farming system on microbial communities in the rhizosphere of maize. Rhizosphere soil samples of two open-pollinated maize populations (“SinPre” and “Pigarro”) cultivated under conventional and organic farming systems were taken during flowering and analyzed by next-generation sequencing (NGS). Phenological data were collected from the replicated field trial. A total of 266 fungi and 317 bacteria genera were identified in “SinPre” and “Pigarro” populations, of which 186 (69.9%) and 277 (87.4%) were shared among them. The microbiota of “Pigarro” showed a significant higher (*P* < 0.05) average abundance than the microbiota of “SinPre.” The farming system had a statistically significant impact (*P* < 0.05) on the soil rhizosphere microbiota, and several fungal and bacterial taxa were found to be farming system-specific. The rhizosphere microbiota diversity in the organic farming system was higher than that in the conventional system for both varieties. The presence of arbuscular mycorrhizae (Glomeromycota) was mainly detected in the microbiota of the “SinPre” population under the organic farming systems and very rare under conventional systems. A detailed metagenome function prediction was performed. At the fungal level, pathotroph–saprotroph and pathotroph–symbiotroph lifestyles were modified by the farming system. For bacterial microbiota, the main functions altered by the farming system were membrane transport, transcription, translation, cell motility, and signal transduction. This study allowed identifying groups of microorganisms known for their role as plant growth-promoting rhizobacteria (PGPR) and with the capacity to improve crop tolerance for stress conditions, allowing to minimize the use of synthetic fertilizers and pesticides. Arbuscular mycorrhizae (phyla Glomeromycota) were among the most important functional groups in the fungal microbiota and *Achromobacter*, *Burkholderia*, *Erwinia*, *Lysinibacillus*, *Paenibacillus*, *Pseudomonas*, and *Stenotrophomonas* in the bacterial microbiota. In this perspective, the potential role of these microorganisms will be explored in future research.

## Introduction

Maize (*Zea mays*) is one of the most important cereal crops in human and animal diets worldwide. Together with rice and wheat, it provides at least 30% of the food calories to more than 4.5 billion people in 94 developing countries (Shiferaw et al., 2011). Aside from providing nutrients for humans and animals, maize serves as a basic raw material to produce starch, oil, protein, alcoholic beverages, food sweeteners, and fuel (Wu and Guclu, 2013).

According to FAOSTAT, from 2012 to 2017, the average world production per year of corn was around 1,036,263,896 t. In Portugal, maize is the most important arable crop, occupying an area of approximately 150,000 ha (ANPROMIS, 2019), producing an annual average of 826,417 Mg from 2012 to 2017 (FAOSTAT, 2019).

Due to new challenges related to the expansion of the human and animal populations allied with the global climatic changes, all opportunities for sustainably increasing the yield are relevant and should be developed. For example, improvements on both agronomic practices and breeding related with microorganism interactions (e.g., control of root diseases) and organic management are appellative proposals since the rhizosphere microbial communities are critically important for soil nitrogen cycling and plant productivity (Schmidt et al., 2016; Emmett et al., 2017; Wille et al., 2018).

In this context, alternatives that promote more resilient farming systems, increasing the economic activity of rural areas as well as preventing the significant loss of biodiversity in these areas, would contribute to more sustainable agriculture. In line with this thought, the traditional varieties are extremely important and should not be neglected (Altieri, 2004) since they present a genetic reservoir that can be used to select for advantages associated with the adaptability and resilience of cultivars to low-input and a permanently changing environment. One of the characteristics of interest in these varieties is the possibility of maintaining genetic diversity through open pollination. New genetic combinations may present new features or capabilities that will allow the plant population to respond with more resilience toward pests, diseases, or even the most adverse weather conditions (Garcia-Tejero et al., 2015). Therefore, traditional varieties can adapt themselves to the environment, having greater adaptability to external factors, increasing the crop population fitness (Altieri and Merrick, 1987; Lane and Jarvis, 2007).

Although traditional Portuguese varieties do not have high yields, they are still cultivated due to their high yield stability even under unfavorable conditions like drought (Garcia-Tejero et al., 2015; Leitão et al., 2019). It is important to note that these varieties also play an important role in the country’s rural economy, especially in the Central and Northern regions of Portugal, as their market value for bread making has increased thanks to their health benefits. This practice is also seen as a viable way to preserve the biodiversity of threatened farming systems (Vaz Patto et al., 2013).

Since 2002, knowledge about microbiota and the rhizosphere has increased exponentially; however, we are just beginning to understand the mechanisms of plant–microorganism interactions (Vandenkoornhuyse et al., 2015; Compant et al., 2019). According to Philippot et al. (2013), the rhizosphere is a hotspot of plant–microbe interactions with a profound influence on plant productivity, and all its functions are extremely important in terms of nutrition, health, and plant quality. Indeed, the rhizosphere is a critical interface that supports the exchange of resources between plants and their related soil environment. It is already known that several plants produce components that interact with the rhizosphere microbiota, thus forming a dynamic structure in which microbial diversity can be modified with soil composition, plant species, different genotypes within the same cultivar, and the development stage of the organism (Turner et al., 2013; Chaparro et al., 2014; Kandel et al., 2017). Moreover, the microbiota can help plants survive climate changes, modify tolerance to abiotic and biotic stresses, affect the plant–pathogen interactions, and change the nutrient contents inside the plant (Chadha et al., 2015). In addition to all these factors, management agricultural practices, the addition of fertilizers, the presence of pathogens, or extreme climatic conditions cause important effects on the microbial diversity composition (Andreote and Silva, 2017). All these factors are highly relevant to improve vigor, growth, and plant’s health (Muller et al., 2016).

In this context, this study aimed to unravel the effect of genotype and farming system on structural diversity and putative functions of the microbial communities in the rhizosphere of two open-pollinated maize populations (“SinPre” and “Pigarro”) cultivated under conventional and organic farming systems in Portugal. With this approach, groups of microorganisms with the potential to modulate the soil quality and fertility were identified and linked to specific conditions, thus potentially contributing to the increasing crop tolerance for stress conditions and to minimize the use of synthetic fertilizers and pesticides.

## Materials and Methods

### Germplasm Characteristics

A synthetic population of maize, “SinPre” (Sintético Precoce), was obtained through the crossing of 12 maize populations (10 Portuguese landraces and two American populations) using a polycross method based on “Nutica” experience (Mendes-Moreira et al., 2009). The 12 maize populations were open-pollinated in geographic isolation (from other maize) in 2009. The border was constituted by equal amounts of seeds of each population. Two equal sets of 12 rows were organized from the earliest to the latest flowering population and *vice versa* for the second set. With this spatial organization, the earliest populations were close to the latest populations. Per row, progenies were submitted to intensive selection among parents during continued cycles. Afterward, a bulk of the best ears was obtained and distributed to farms in different agroclimatic regions of Portugal. The purpose of this new synthetic population was to provide farmers with a new population, with high diversity that could be adapted to their needs, so that farmers can select the characteristics they appreciate the most (P. Mendes-Moreira, October 2019, personal communication). “Pigarro” is a Portuguese population used to produce maize bread that presents a strong emergency vigor, great tolerance at low temperatures, early flowering and fast drying of the grain, and with ripening group FAO 300. It has white and flint kernels and its ear is known for the high number of kernels per row (between 18 and 28) as well as a strong expression of fasciation (Mendes-Moreira et al., 2017).

### Field Characterization and Agronomical Practices

Field trials were established at two locations in Coimbra, Portugal, that belong to Coimbra College of Agriculture: “Caldeirão” in organic (40°130′0.22″N, 8°260′47.69″W) and “Vagem grande” in conventional (40°13′16.2″N, 8°28′29.3″W), with a distance of approximately 2.4 km. Both locations have alluvial soils with a medium field texture; however, they can be differentiated between organic and conventional in the organic matter (1.8 vs. 0.8%), pH (6.4 vs. 6.7), available phosphorus (high vs. very high), and available potassium (very high vs. high) (Supplementary Table 1).

The maize seed used was produced in the organic location in 2018 and seeds were not treated. For both fields, the preceding crop was maize.

In conventional agriculture soil, tillage started on 15/05/2019 followed by the first fertilization on 21/05/2019. The fertilizer was distributed (12:20:12 NPK, 318 kg/ha) with a centrifugal fertilizer spreader and incorporated with a rotor tiller. On 24/05/2019, the fertilizer (12:20:12 NPK) was simultaneously incorporated at a rate of 592 kg/ha during sowing. Sowing was conducted with a single-seed sowing machine with nine plants per square meter, followed by a herbicide application (609.38 g/ha terbuthylazine + 121.88 g/ha mesotrione + 1,015.63 g/ha *S*-metolachlor). On 25/06/2019, a fertilizer application of 560 kg/ha with a composition of 40% nitrogen (N) and 14% sulfur (SO_3_) was done, followed by the application of herbicides (25.13 g/ha nicossulfuron + 375 g/ha terbuthylazine + 165 g/ha sulcotrione). Finally, a pilling up through mechanical weed control with a harrow helped to control weeds.

In organic and low-input farming, soil tillage was done on 13/05/2019. Sowing occurred on 14/05/2019. Sowing was conducted with a single-seed sowing machine with six plants per square meter. Weed control was carried out manually since sowing, until late June, when pilling up was finally done. No fertilizer was applied.

### Germplasm Characterization

For 20 randomly chosen plants from each population, an adaptation of the HUNTERS descriptor was used based on the field data collected during the monitoring of the maize crop: height (*H*), height of first ear insertion (H1E), uniformity (*U*), root (*R*%), and stalk lodging percentage (*S*%). IBMSPSS^®^ statistics program was used for phenotyping data analyses (IBM Corp, 2020).

### Sampling of Maize Rhizosphere

The soil was collected at the flowering stage in the two maize populations: “SinPre” and “Pigarro”. For each population, samples were collected from the organic and conventional farming systems. Within each plot, three individual plants, separated by at least 5 m from each other, were selected. Entire plants were dug up with a soil monolith in the middle of them. Bulk soil was taken off the plant roots by vigorous shaking. Plant fine roots were collected from each plant, stored in cool temperature, and moved rapidly to the laboratory where rhizospheric soil samples (1–2 mm soil adhering to roots) were collected.

### DNA Extraction and Sequencing

Total DNA was extracted using Nucleospin Soil Kit (Macherey Nagel, Düren, Germany) with Buffer SL1 in combination with Enhancer SX, according to manufacturer’s instructions. Internal transcribed spacer 2 (ITS2) region amplicon libraries and Illumina 16S ribosomal RNA (rRNA) genes were generated and sequenced at Genoinseq (Portugal). The DNA was amplified for the hypervariable regions with specific primers and further reamplified in a limited-cycle PCR reaction to add sequencing adapters and dual indexes. The first PCR reactions were performed using a pool of forward primers—ITS3NGS1_F-5′-CATCGATGAAGAACGCAG-3′, IT S3NGS2_F-5′-CAACGATGAAGAACGCAG-3′, ITS3NGS3_F-5′-CACCGATGAAGAACGCAG-3′, ITS3NGS4_F-5′-CATCG ATGAAGAACGTAG-3′, ITS3NGS5_F-5′-CATCGATGAAGA ACGTGG-3′, ITS3NGS10_F-5′-CATCGATGAAGAACGCTG-3′—and reverse primer ITS4NGS001_R-5′-TCCTSCGCTTA TTGATATGC-3′ for fungi (Tedersoo et al., 2014) and forward primer Bakt_341F-5′-CCTACGGGNGGCWGCAG-3′ and reverse primer Bakt_805R-5′-GACTACHVGGGTATCTAATCC-3′ for bacteria (Herlemann et al., 2011; Klindworth et al., 2013). The second PCR reaction added indexes and sequencing adapters to both ends of the amplified target region according to the manufacturer’s recommendations (Illumina, 2013). Negative PCR controls were included for all amplification procedures. PCR products were then one-step purified and normalized using a SequalPrep 96-well plate kit (Thermo Fisher Scientific, Waltham, United States) (Comeau et al., 2017), pooled, and pair-end sequenced in the Illumina MiSeq^®^ sequencer with the V3 chemistry, according to the manufacturer’s instructions (Illumina, San Diego, CA, United States) at Genoinseq (Cantanhede, Portugal).

### *In silico* Functional Analysis

Prediction of functional bacterial and fungal diversity within the 16S rRNA and ITS2 sequence libraries was performed using PICRUSt (Langille et al., 2013) and FUNGuild (Nguyen et al., 2015), respectively. PICRUSt predicts the potential metagenomic gene content of a 16S amplicon library based on genomic information of the bacteria represented within the Greengenes 16S database. To perform the process within the PICRUSt program, samples derived from the QIIME2 process, before taxonomic assignment, were selected and grouped into 97% operational taxonomic units (OTUs) against the Greengenes database v.13.8. The nearest sequence taxon index (NSTI) within the PICRUSt pipeline was also calculated as a quality control to validate the accuracy of the predicted functional annotations. FUNGuild assigns trophic modes to fungal taxa based on a comparison to a curated database of fungal lifestyles (*sensu* Tedersoo et al., 2014): pathotroph, symbiotroph, and saprotroph. Trophic mode refers to the mechanisms through which organisms obtain resources, providing putative information on the ecology of such organisms (Nguyen et al., 2015). Functional assignments through FUNGuild are based on taxonomy and are possible only if the taxa have been classified at the genus level or if the taxa belong to a fungal group with an exclusive lifestyle. Input data for FUNGuild was the OTU table.

### Statistical and Bioinformatics Analysis

Raw reads were extracted from the IlluminaMiSeq^®^ System in fastq format and quality-filtered with PRINSEQ version 0.20.4 to remove sequencing adapters, reads with less than 100 bases for the ITS2 region and 150 bases for the 16S rRNA gene, and trim bases with an average quality lower than Q25 in a window of 5 bases (Schmieder and Edwards, 2011). The forward and reverse reads were merged by overlapping paired-end reads with Adapter Removal version 2.1.5 using default parameters (Schubert et al., 2016). After sequencing, the bacterial and fungal communities were analyzed using the QIIME software package. Chimeric sequences were removed using the consensus method and clustered in OTUs at 99% using a reference. Taxonomy was assigned to bacterial and fungal OTU sequences using Greengenes v13.8 and UNITEv.7.2, respectively. The phylogenetic classification was performed to the genus level. The rarefaction curves obtained were saturated for each sample, demonstrating that the OTUs recovered were representative of the bacterial and fungi diversity, supporting a robust analysis.

The alpha diversity indexes Shannon index (*H*′), Simpson (*D*), and Chao1 were calculated with the Phyloseq package to include in MicrobiomeAnalyst (Dhariwal et al., 2017). The statistical significance of grouping based on experimental factor was estimated using *t*-test/analysis of variance (ANOVA, *P* < 0.05) to determine differences in the alpha diversity indexes among variables: “SinPre” and “Pigarro” populations and conventional and organic farming systems. A non-supervised principal component analysis (PCA) was performed to compare the bacterial and fungal community structures. Statistical analyses were performed with ANOVA at *P* < 0.05 using R software v.4.0. Venn diagrams were generated with Venny 2.1 (Oliveros, 2007–2015) to identify shared and unique taxa of each population according to the farming system. To identify fungal and bacterial taxa that differed in the relative abundance among population genotypes and farming systems in the rhizosphere of maize, a linear discriminant analysis (LDA) was performed combined with effect size (LEfSe) using a graphical interface in Galaxy version 1.0 (The Huttenhower Lab, 2018). A *P*-value of < 0.05 and a score ≥ 2.0 were considered significant in Kruskal–Wallis and pairwise Wilcoxon tests, respectively.

## Results

### Germplasm Agronomic Characterization

The averages of the data collected in the field according to the HUNTERS (Mendes-Moreira et al., 2017) descriptor for both populations (“SinPre” and “Pigarro”) in conventional and organic farming are detailed in Supplementary Table 2. The phenotypic characterization indicates that “SinPre” in conventional farming was significantly higher than in organic farming for plant and first ear height, but no differences were observed for “Pigarro.” Within farming systems (conventional and organic), there were no significant differences between the tested populations (“SinPre” × “Pigarro”) for the measured parameters. Both populations showed values of uniformity between 2 and 4, and for the parameters angle (*N*), tassel (*T*), and ear insertion position (*E*), the values variated from 5 to 6. Root lodging (*R*) ranged from 0% (“SinPre” conventional) to 4.1% (“Pigarro” conventional). Stalk lodging (*S*) ranged from 10.5% (“Pigarro” organic) to 17.1% (“SinPre” conventional).

### Fungal and Bacterial Rhizosphere Microbiota Associated With Two Maize Populations

The structural compositions of the fungi and bacterial communities associated with the rhizosphere soils of two traditional populations of maize grown were grouped into 973 fungal and 4,051 bacterial OTUs.

**FIGURE 1:**
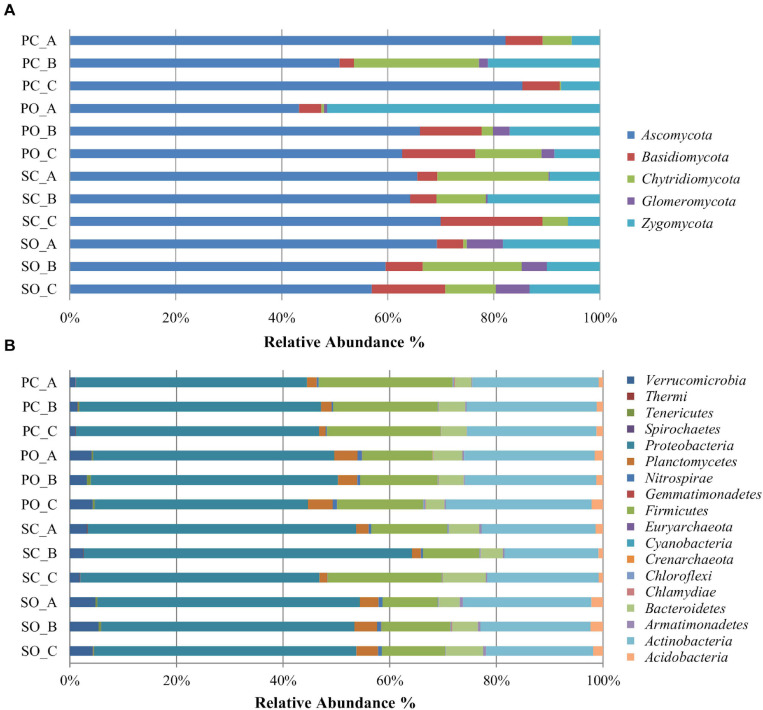
Relative abundance of different fungal **(A)** and bacterial **(B)** phyla in the soil rhizosphere in both populations. *PC_A*, “Pigarro” conventional plant A; *PC_B*, “Pigarro” conventional plant B; *PC_C*, “Pigarro” conventional plant C; *PO_A*, “Pigarro” organic plant A; *PO_B*, “Pigarro” organic plant B; *PO_C*, “Pigarro” organic plant C; *SC_A*, “SinPre” conventional plant A; *SC_B*, “SinPre” conventional plant B; *SC_C*, “SinPre” conventional plant C; *SO_A*, “SinPre” organic plant A; *SO_B*, “SinPre” organic plant B; *SO_C*, “SinPre” organic plant C.

**FIGURE 2:**
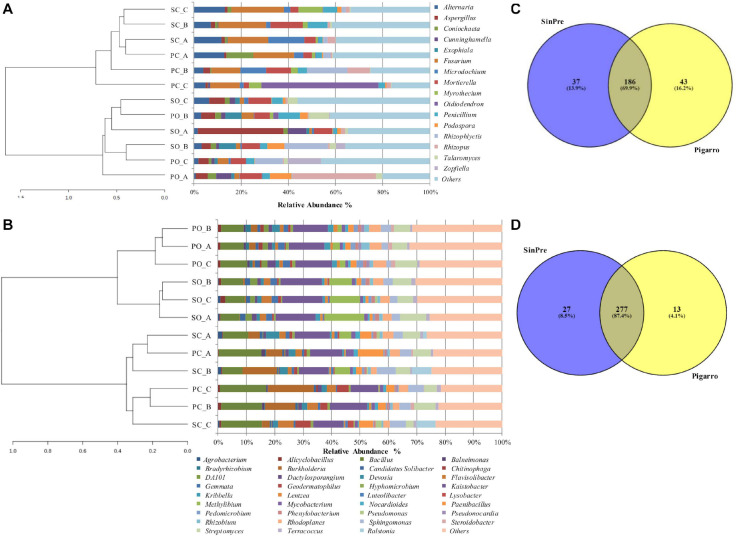
Relative abundance of different fungal **(A)** and bacterial **(B)** genera in the soil rhizosphere in both populations representing the genera showing more than 1% relative abundance of all reads. The genera representing less than 1% of the total reads are grouped into “Others.” Venn diagrams showing the common and exclusive fungal **(C)** and bacterial **(D)** genera of the rhizosphere of the maize populations “SinPre” and “Pigarro.” *PC_A*, “Pigarro” conventional plant A; *PC_B*, “Pigarro” conventional plant B; *PC_C*, “Pigarro” conventional plant C; *PO_A*, “Pigarro” organic plant A; *PO_B*, “Pigarro” organic plant B; *PO_C*, “Pigarro” organic plant C; *SC_A*, “SinPre” conventional plant A; *SC_B*, “SinPre” conventional plant B; *SC_C*, “SinPre” conventional plant C; *SO_A*, “SinPre” organic plant A; *SO_B*, “SinPre” organic plant B; *SO_C*, “SinPre” organic plant C.

A total of five fungal phyla, 25 classes, 79, orders, 150 families, and 266 genera were identified (Supplementary Table 3). The most abundant and diverse phylum was Ascomycota (64%, comprising 144 genera), followed by Zygomycota (18%, comprising 12 genera), Basidiomycota (8%, comprising 87 genera), Chytridiomycota (8%, comprising 11 genera), and Glomeromycota (2% comprising 12 genera) (Figure 1A). The most abundant genera within Ascomycota were *Odiodendron* (13%), *Fusarium* (12%), and *Aspergillus* (11%); within Basidiomycota were *Rhodotorula* (18%), *Puccinia* (13%) *Papiloterma* (12%), and *Conocybe* (9%); within Chytridiomycota the genera *Rhizoplictis* (65%), *Olpidium* (17%), and *Powellomyces* (8%); within Glomeromycota were *Dentiscutata* (45%) *Gigaspora* (22%), and *Paraglomus* (15%); and within Zygomycota were *Mortierella* (39%), *Rhizopus* (39%), and *Cunninghamella* (13%) (Figure 2A). Overall, the most abundant genera were *Oidiodendron* and *Fusarium* (with 8% each), *Rhizopus*, *Morteriella*, and *Aspergillus* (with 7% each), *Rhizophlyctis* (5%), *Alternaria* and *Penicillium* (with 4% each), *Podospora* and *Microdochium* (with 3% each), and *Cunninghamella*, *Coniochaeta*, *Exophiala*, *Talaromyces*, *Myrothecium*, and *Zopfiella* (with 2% each), accounting for approximately 68% of the total diversity. Thirty-seven (14%) genera were shared by all samples; nevertheless, 22% of these bacterial taxa were rare since each represented less than 1% of the total diversity (Supplementary Table 3).

For the bacterial microbiota, a total of 18 phyla, 41 class, 72 orders, 158 families, and 317 genera were identified (Supplementary Table 4). The most abundant and diverse bacterial phylum was Proteobacteria (47%, comprising 119 genera), followed by Actinobacteria (23%, comprising 83 genera), Firmicutes (16%, comprising 59 genera), and Bacteroidetes (5%, comprising 36 genera) (Figure 1B). At the genus level, the most abundant by phylum were *Kaistobacter* (26%), *Burkholderia* (10%), *Methylibium* (9%), *Sphingomonas* (8%), and *Rhodoplanes* (7%) within Proteobacteria; *Streptomyces* (26%), *Geodermatophilus* (7%), and *Mycobacterium* (6%) within Actinobacteria; *Bacillus* (65%), *Paenibacillus* (18%), and *Alicylobacillus* (5%) within Firmicutes; and *Flavisolibacter* (51%) and *Chitinophaga* (14%) within Bacteroidetes (Figure 2B). Overall, the most abundant genera were *Kaistobacter* (12%), *Bacillus* (10%), *Streptomyces* (6%), *Burkholderia* (5%), *Methylibium* and *Sphingomonas* (with 4% each), *Rhodoplanes*, *Paenibacillus*, and *Flavisolibacter* (with 3% each), and *Devosia*, *Geodermatophilus*, and *Gemmata* (with 2% each), accounting for approximately 55% of the total diversity. One hundred and thirteen (36%) genera were shared by all samples, while only 20 of these bacterial taxa were restricted to one sample (Supplementary Table 4).

### Core Rhizosphere Microbiota Associated With Different Maize Populations

The rhizosphere of the maize populations showed specific fungal and bacterial OTUs for each population and a cluster of shared OTUs. Comparing the fungal and bacterial microbiota of the “Pigarro” and “SinPre” populations, 69.9 and 87.4% of the fungal and bacterial genera, respectively, were shared between populations, demonstrating the existence of a “core” maize phylogeny (Figures 2C,D).

Eighty-seven fungal genera composed the core fungal rhizosphere microbiota, affiliated with Ascomycota (54 genera), Basidiomycota (20 genera), Chytridiomycota (three genera), Glomeromycota (three genera), and Zygomycota (seven genera) (Supplementary Table 5).

The core rhizosphere bacterial microbiota present in both maize populations was composed of 183 genera, affiliated with Proteobacteria (64 genera), followed by Actinobacteria (54 genera), Firmicutes (22 genera), Bacteroidetes (17 genera), Verrumicrobia (10 genera), Planctomycetes (four genera), Chlamydiae (three genera), Acidobacteria (three genera), Chloroflexi (two genera), and Armatimonadetes, Crenarchaeota, Nitrospirae, and Thermi, with one genus each (Supplementary Table 5).

Specific fungal genera were only associated with the “SinPre” population (37 genera, corresponding to 13.9%) and with the “Pigarro” population (43 genera, corresponding to 16.2%). Regarding the bacterial microbiota, 13 genera (4.1%) were exclusive to the “SinPre” population and 27 to the “Pigarro” population (8.5%) (Figures 2C,D). The population-specific genera are shown in Supplementary Table 6.

**FIGURE 3:**
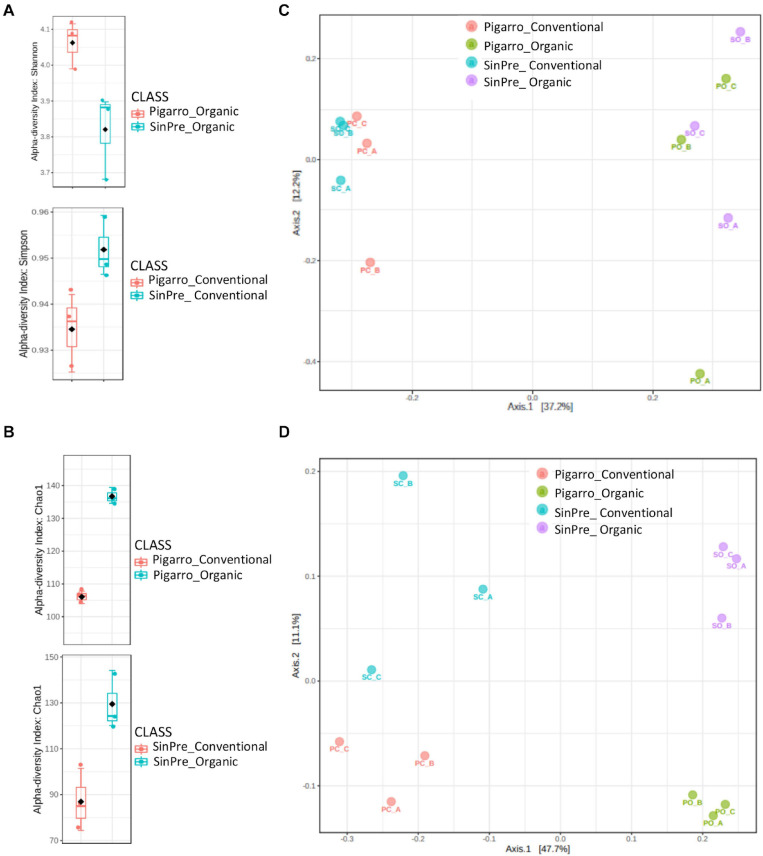
Box plot illustrating statistically significant α diversity measures of the effect of farming system in fungal **(A)** and bacterial **(B)** communities in the maize rhizosphere. Principal coordinate analysis (PCoA) was performed using the prcomp package to illustrate the effect of farming system on the fungal **(C)** and bacterial **(D)** communities in the maize rhizosphere. The calculation is based on singular value decomposition of the fungal and bacterial communities for the farming system. A greater distance between the two samples indicates low similarity. The percentage of variation is explained by component 1 (PC1) and by component 2 (PC2).

Fungal communities of the rhizospheric soil samples did not differ between populations. Bacterial communities were only significantly different in the rhizosphere associated with the “Pigarro” genotype in the organic farming system (*F* = 3.5, *P* < 0.05). The maize genotype was a significant factor structuring the bacterial community in both farming systems, supported by Shannon (*t*-test = 3.0627, *P* < 0.05) and Simpson (*t*-test = −2.7668, *P* < 0.05) α diversity indexes (Figures 3A,B and Table 1). Principal coordinate analysis (PCoA) further evidenced that most of the variations on the dataset could be attributed to the population genotype (Figures 3C,D).

**TABLE 1:** Experimental factors predicting α diversity of the rhizosphere-associated fungal and bacterial communities in maize.

		Chao1		Shannon		Simpson	
		*t*-test	*P*	*t-*test	*P*	*t*-test	*P*
**Fungi**							

Genotype	PC_PO	−17.014	**0.0001**	−1.5369	0.1992	−0.8259	0.4663
	SC_SO	−3.939	**0.0171**	−2.1054	0.1503	−0.4810	0.6759
Farming system	PC_SC	2.4068	0.1330	−0.5873	0.6123	−0.6863	0.5625
	PO_SO	0.4327	0.9591	−0.1506	0.8880	0.0035	0.9974

**Bacteria**							

Genotype	PC_SC	0.3649	0.7338	−2.1922	0.1001	−2.7668	0.0540*
	PO_SO	0.9858	0.3956	3.0627	0.0530*	2.8629	0.0623
Farming system	PC_PO	−0.48906	0.6513	−7.3396	**0.0036**	−5.1265	**0.0171**
	SC_SO	−0.20527	0.8150	−0.50067	0.6437	0.5229	0.6288

*P, “Pigarro”; S, “SinPre”; C, conventional; O, organic. Statistics describe the linear random intercept models of Shannon and Simpson diversity and Chao1 richness in the rhizosphere. Alpha diversity analysis was performed using the phyloseq package. The statistical significance of grouping based on the experimental factor was estimated using t-test/ANOVA. Bold values indicate statistically significant results (P < 0.05). ^∗^Statistically significant.*

**FIGURE 4:**
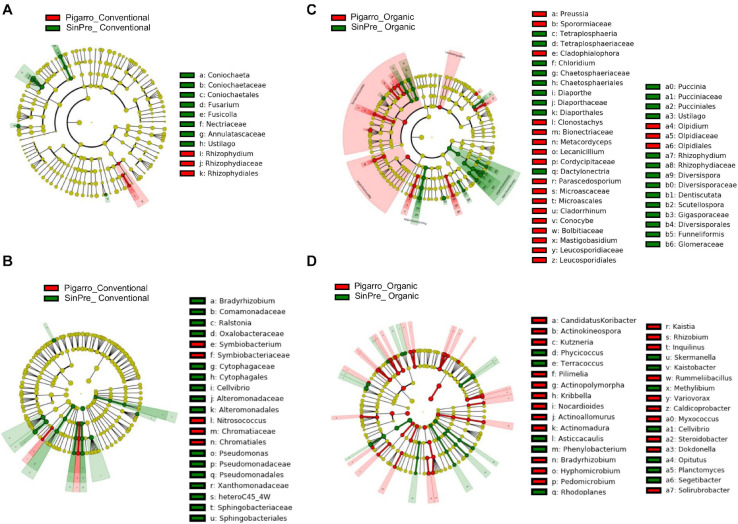
Linear discriminant analysis (LDA) combined with effect size (LEfSe) was used to identify the most differentially abundant taxa among the population genotypes in the rhizosphere of maize. Cladogram generated by LEfSe indicating differences of fungi **(A,B)** and bacteria **(C,D)** at the phylum, class, family, and genus levels (relative abundance, ≤0.5%). Each successive *circle* represents a phylogenetic level. The *red* and *green circles* mean that “Pigarro” conventional and organic (*red*) and “SinPre” conventional and organic (*green*) showed differences in relative abundance; *yellow circles* mean non-significant differences. Differing taxa are listed on the *right side of the cladogram*. Bar graph showing the LDA scores for bacteria is represented in Supplementary Figure 1. Only taxa meeting an LDA significant threshold >2 are shown.

The LEfSe detected 64 fungal (12 PC_SC and 52 PO_SO) and 98 bacterial (24 PC_SC and 74 PO_SO) bacterial clades in the rhizosphere, which discriminated the microbial communities between populations (Figure 4). Annulatascaceae, Coniochaetaceae, Nectriaceae (class Sordariomycetes) were the major fungi families that contributed to differentiate the fungal communities associated with “Pigarro” and Rhizophydiaceae (class Chytridiomycetes) with “SinPre” under the conventional farming system. Under the organic farming system, the differences between genotypes were more pronounced due to the contributions of several other classes, namely, Agaricomycetes, Eurotiomycetes, Leotiomycetes, and Microbotryomycetes in “Pigarro” and Dothideomycetes, Glomeromycetes, Pucciniomycetes, and Ustilaginomycetes in “SinPre” (Figure 4A).

The main bacterial families that contributed to differentiate the communities associated with population genotypes in the conventional agricultural system were Alteromonadaceae, Bradyrhizobiaceae, Comamonadaceae, Cytophagaceae, Oxalobacteraceae, Pseudomonadaceae, Sphingobacteriaceae, and Xanthomonadaceae in “SinPre” and Chromatiaceae and Symbiobacteriaceae in “Pigarro.” The differences between populations were more pronounced in the organic farming system because of the presence of members of the classes Alcaligenaceae Caulobacteraceae, Cellvibrionaceae, Chitinophagaceae, Intrasporangiaceae, Hyphomicrobiaceae, Opitutaceae, Planctomycetaceae, Rhodospirillaceae, and Sphingomonadaceae in “SinPre” and Acidobacteriaceae, Bradyrhizobiaceae, Caldicoprobacteraceae, Comamonadaceae, Cystobacterineae, Hyphomicrobiaceae, Micromonosporaceae, Nocardioidaceae, Nocardiopsaceae, Planococcaceae, Pseudonocardiaceae, Rhizobiaceae, Rhodanobacteraceae, Rhodospirillaceae, Solirubrobacteraceae, and Steroidobacteraceae in “Pigarro” (Figure 4B). Detailed information can be found in Supplementary Figure 1. Several genera were significantly different between population genotypes, as detailed in Supplementary Table 7.

Specific fungal and bacterial OTUs associated with the “SinPre” population ranged from 9.4 and 13.8% in the conventional and between 45.3 and 17.2% in the organic farming system, respectively. The core microbiota of the “SinPre” population corresponded to 45.3% of the fungal and 69% of bacterial total diversity. A similar trend was observed in the dataset from the “Pigarro” population, with specific fungal and bacterial OTUs comprising 12.7 and 11.8% in conventional and 39.3 and 18.4% in the organic farming system, respectively. The core microbiota of the “Pigarro” population corresponded to 48% of the fungi and 69.7% of the bacteria total diversity. The farming system-specific genera are shown in Supplementary Table 8.

### Core Rhizosphere Microbiota Associated With Farming Systems

The results obtained showed that rhizospheric soil harbored a distinct microbiota according to the farming system (*P* < 0.05; Table 1). This was visible in the cluster-based analysis of the fungal and bacterial community structure and composition, where maize populations were clustered by the farming system (Figure 2). Indeed, the rhizosphere showed specific fungal and bacterial OTUs for each farming type and a cluster of shared OTUs. Comparing the total dataset of the fungal and bacterial microbiota of the conventional and organic farming systems, 49.2 and 74.8% of fungal and bacterial OTUs, respectively, were shared between the two systems. Specific OTUs were associated with the conventional farming system, 25 fungal and 28 bacterial genera, representing 9.4 and 8.8% of the community, respectively. Regarding the organic farming system, the relative abundance of specific fungal (110, 41.4%) and bacterial (52, 16.4%) OTUs was considerably higher.

### Core Rhizosphere Microbiota Across Both Maize Populations

The farming system was a significant factor structuring the fungal community predicted by Chao1 diversity in the “Pigarro” (*t*-test = −17 014, *P* < 0.05) and “SinPre” (*t*-test = −3,939, *P* < 0.05) genotypes (Figure 3A and Table 1). Besides, the mycobiota associated with “SinPre” was significantly different between the farming systems (*F* = 9.08, *P* < 0.05). However, the farming systems had a much weaker influence on the rhizosphere-associated bacterial communities. The PCoA further evidenced that most of the variations on the dataset could be related to the farming system (Figures 3C,D).

At the phylum level, the impact of the farming system on fungal diversity associated with the “Pigarro” rhizosphere translated into a higher dominance of Ascomycota in the conventional system, partially replacing the Zygomycota in the organic system. In the “SinPre” population, the relative abundance of Ascomycota was similar in both farming systems. Importantly, a notably higher presence of mycorrhizae (Glomeromycota) was observed in both populations cultivated in the organic farming system, in clear contrast to the conventional farming system (Figure 1A).

**FIGURE 5:**
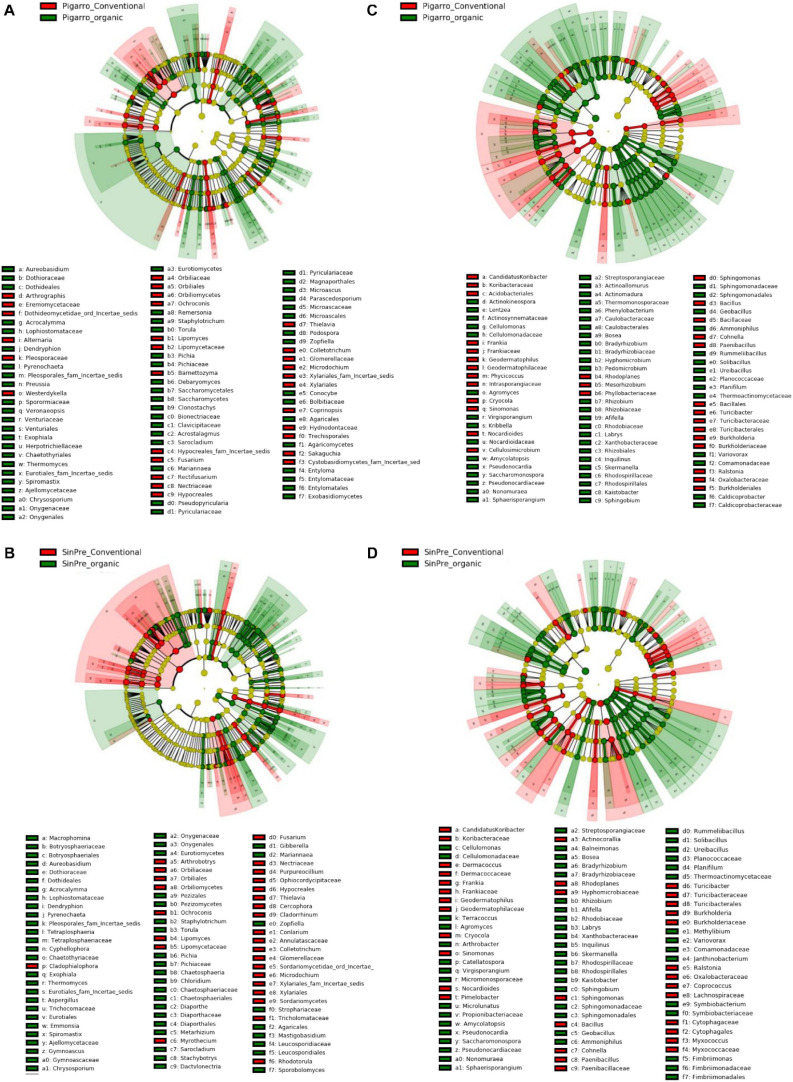
Linear discriminant analysis (LDA) combined with effect size (LEfSe) was used to identify the most differentially abundant taxa among farming systems in the rhizosphere of maize. Cladogram generated by LEfSe indicating differences of fungi **(A,B)** and bacteria **(C,D)** at the phylum, class, family, and genus levels (relative abundance, ≤0.5%). Each *successive circle* represents a phylogenetic level. The *red* and *green circles* mean that conventional (*red*) and organic (*green*) showed differences in relative abundance; *yellow circles* mean non-significant differences. Differing taxa are listed on the *right side of the cladogram*. Bar graph showing the LDA scores for bacteria is represented in Supplementary Figure 1. Only taxa meeting an LDA significant threshold >2 are shown.

The LEfSe detected 255 fungal (124 PC_PO and 131 SC_SO) and 266 bacterial (138 PC_PO and 128 SO_SC) clades in the rhizosphere, which discriminated the microbial communities between the farming systems (Figure 5). Dothideomycetes, Orbiliomycetes, Sordariomycetes, and Tremellomycetes were the major fungi classes that contributed to differentiate the fungal communities associated with the conventional farming system despite the considered population, while under the organic farming system the differences were due to the contribution of several other classes, namely, Eurotiomycetes, Mortierellomycetes, Saccharomycetes, and Ustilaginomycetes. Worth noticing is that Glomeromycetes and Pucciniomycetes were highly abundant in the “SinPre” population cropped in organic farming (Figures 5A,B). The main bacterial classes that contributed to differentiate the communities associated with farming systems were Bacilli and Verrucomicrobiae in the conventional farming system and Alphaproteobacteria, Anaerolineae, Fimbriimonadia, Mollicutes, Nitrospira, Opitutae, Planctomycetia, Spartobacteria, and Solibacteres in the organic farming system (Figures 5C,D).

### *In silico* Metagenome Analysis

**FIGURE 6:**
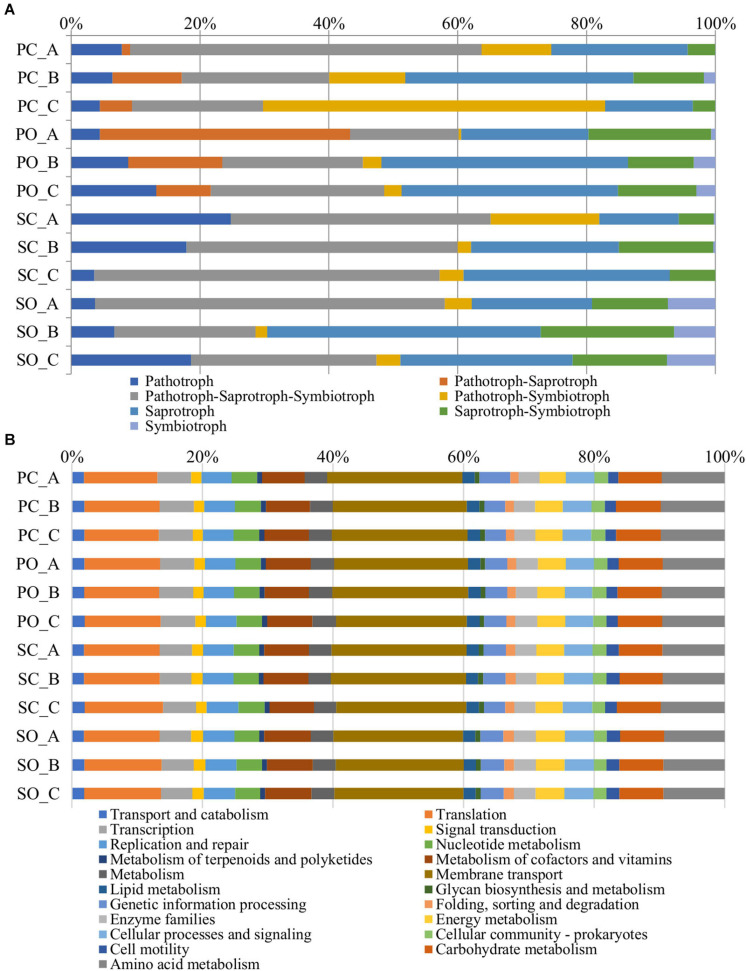
Metagenome predicted functions classified using FUNGuild for fungi **(A)** and the Kyoto Encyclopedia of Genes and Genomes (KEGG) level 2 database in PICRUSt software for bacteria **(B)** showing the most abundant functions throughout the rhizospheric soil samples.

The functional predictions at the fungal level in both populations and both farming systems are shown in Figure 6A. An increase in the trophic modes defined as pathotroph–saprotroph, saprotroph-symbiotroph, and symbiotroph was observed in the “Pigarro” population under the organic farming system. The same population in the conventional farming system experienced an increase in the pathotroph–saprotroph–symbiotroph and pathotroph–symbiotroph trophic modes. In the “SinPre” population under the organic farming system, the trophic modes defined as pathotroph–saprotroph–symbiotroph, saprotroph, saprotroph–symbiotroph, and symbiotroph were more abundant, while the latter was practically non-existent in the conventional farming system.

In detail, the trophic mode pathotroph comprised 32 genera of plant pathogens. In detail, 26 were detected in “Pigarro,” five specific to the conventional farming system (*Chalastospora*, *Curvularia*, *Lectera*, *Rhizophydium*, and *Volutella*) and 10 specific to the organic farming system (*Cercospora*, *Kochiomyces*, *Dactylaria*, *Entyloma*, *Macrophomina*, *Sporisorium*, *Ramularia*, *Sclerotinia*, *Thanatephorus*, and *Gjaerumia*), while the remaining 11 were shared between farming systems (*Dendryphion*, *Farysia*, *Ilyonectria*, *Olpidium*, *Anthracocystis*, *Clonostachys*, *Drechslera*, *Gibberella*, *Monographella*, *Powellomyces*, and *Puccinia*). Similarly, in the “SinPre” population, 32 genera of fungal plant pathogens were present, three specific to the conventional farming system (*Olpidium*, *Lectera*, and *Thanatephorus*), 13 related to the organic farming system (*Protomyces*, *Dendryphion*, *Rhizophydium*, *Exserohilum*, *Macrophomina*, *Sphacelotheca*, *Entyloma*, *Microstroma*, *Glomosporium*, *Sporisorium*, *Taphrina*, *Ilyonectria*, and *Ramularia*), and eight shared between farming systems (*Monographella*, *Puccinia*, *Powellomyces*, *Clonostachys*, *Gibberella*, *Curvularia*, *Anthracocystis*, and *Drechslera*). Both populations shared seven plant pathogens despite the farming system: *Monographella*, *Puccinia*, *Powellomyces*, *Clonostachys*, *Gibberella*, *Anthracocystis*, and *Drechslera*. Another important observation from the dataset was the increase in abundance and diversity of arbuscular mycorrhiza in the organic farming system.

A total of 6,886 functional orthologs were predicted to be present in the analyzed bacterial communities, corresponding to 21 level 2 Kyoto Encyclopedia of Genes and Genomes (KEGG) pathways (Figure 6B). The results showed nine pathways for metabolism, four for genetic information processing and unclassified, three pathways for cellular processes, and two for environmental information processing. The average relative abundance of the metabolism function was 33.95%, followed by genetic information processing (21.13%), environmental information processing (20.37%), cellular processes (5.34%), and unassigned with 19.22%. The most abundant KEGG pathway belonged to membrane transport, followed by translation, amino acid metabolism, carbohydrate metabolism, and metabolism of cofactors and vitamins.

## Discussion

The rhizosphere is a specialized region where interactions between plant roots and the surrounding soil-associated microorganisms take place. Rhizospheric soil differs from bulk soil not only due to the direct effect of these microorganisms but also because root growth modifies the composition of the soil. Roots can release rhizodeposits, a wide range of substances containing carbon (e.g., root cells, mucilage, volatiles, and exudates), selecting and enhancing groups of microorganisms (Harkes et al., 2019), which, in addition to modifying some soil characteristics, can play a role in the plant health status.

This work intends to characterize the structural compositions of fungal and bacterial communities present in the rhizospheric soil associated with two maize populations cultivated in different farming systems. Although it is difficult to draw robust conclusions, significant interactions between the microbial diversity, farming systems, and genotype were obtained. Moreover, our results are in line with previous studies (Granzow et al., 2017; Ginnan et al., 2018; Berlanas et al., 2019) including those on maize crop (Galazka and Grzadziel, 2018; Brisson et al., 2019; Kong et al., 2020), shedding some light into the potential beneficial impacts of maize landraces and organic cultivation techniques into the rhizosphere microbial communities and network assembly.

### Impact of Maize Populations and Farming System on the Associated Rhizosphere Mycobiota

The microbial community analysis revealed significant differences in the relative abundance of OTUs between maize populations, evidencing that plant genotype has a significant effect on the structure of the rhizosphere-associated microbiota. In our dataset, a higher portion of the differentially selected microbial community by genotypes was discovered compared with previous studies (Brisson et al., 2019). Indeed, 30.1% of the fungal community and 12.6% of the bacterial community were significantly associated with a specific population.

The novel landrace “SinPre” was derived from 12 populations, while the traditional landrace “Pigarro” encompassed less diversity. We expected that this intraspecific diversity would be translated into more heterogeneous rhizosphere mycobiota, but this was not the case. The rhizosphere mycobiota associated with the “Pigarro” and “SinPre” populations were similar, but significantly influenced by the farming systems. A higher heterogenicity was found in the rhizosphere mycobiota under the organic farming system, in line with previous works (Hartmann et al., 2015; Figure 3C). Also, high fertilization regimes are known to select and enrich fungi populations (Enebe and Babalola, 2020), aiding to explain the previously described diversity.

The fungal community was dominated by the Ascomycota phylum, which agrees with previous studies on the maize rhizosphere (Galazka and Grzadziel, 2018). The impact of the farming system in fungal diversity associated with the “Pigarro” rhizosphere translated into a higher dominance of Ascomycota in the conventional system, replaced in the organic system by Zygomycota. Our results follow the study by Galazka and Grzadziel (2018), where the abundance of the order Hypocreales (Ascomycota) was associated with a conventional farming system. Moreover, Hypocreales, Sordariales, and Helotiales were among the most abundant classes in our dataset, similar to the results described by Klaubauf et al. (2010) on the fungal diversity in arable soils. On the contrary, our results did not support the previously described relationship between the order Sordariales and the conventional farming system since, in our dataset, it was more abundant in the organic farming system.

This study also showed that the farming system was a significant factor structuring the maize-associated fungal community. This influence was less pronounced in the associated bacterial community. A similar trend was described by Galazka and Grzadziel (2018) in a study combining different cultivation practices, reinforcing the impact that these practices have on the microbial community and soil function. Our results showed that rhizospheric soil harbored a distinct mycobiota according to the farming system. Dothideomycetes, Orbiliomycetes, Sordariomycetes, and Tremellomycetes were the main classes within the Ascomycota phylum that contributed to differentiate the fungal communities associated with the conventional agricultural system. The Dothideomycetes class includes several endophytic and plant saprobic taxa, as well as crop-related pathogens. Both *Alternaria* sp. and *Cladosporium* sp. are examples of the latter that were associated with the conventional farming in our dataset. The class Orbiliomycetes includes most of the known nematode-trapping fungi constituting an important part of the subsoil ecosystem, among which *Arthrobotrys* sp. was associated in this study with conventional farming. *Fusarium* was the most abundant genus of the class Sordariomycetes and has been commonly associated with the maize rhizosphere. This genus includes saprotrophic and plant pathogenic species causing significant economic losses in cereals, especially in maize (McMullen et al., 2012; Salgado et al., 2015). Also, *Fusarium* can produce phytotoxins that inhibit the growth of infected plants and can act as virulence factors (Salgado et al., 2015). Tremellomycetes belong to Basidiomycota, the second most abundant phylum of our dataset, including *Cryptococcus* sp., known for the ability to aid plants to extract nutrients from the soil (Wolf et al., 2014).

Classes Eurotiomycetes, Mortierellomycetes, Saccharomycetes, Ustilaginomycetes, Glomeromycetes, and Pucciniomycetes shaped the rhizospheric mycobiota associated with maize cropped in the organic system. Interestingly, the latter two were more abundant in “SinPre” than in the “Pigarro” maize population. Eurotiomycetes and Saccharomycetes belong to Ascomycota, including two of the most important and problematic genera in the cultivation of maize, *Penicillium* and *Aspergillus*, present in our dataset. *Penicillium* species are ubiquitous in the soil wherever organic material is available (Duniere et al., 2017), while *Aspergillus* sp., in spite being involved in lignin degradation and (or) melanin synthesis (Levasseur et al., 2008), crucial to improving the amount of organic matter available in the soil, can also produce the mycotoxin deoxynivalenol (DON), which is harmful to humans and livestock. Ustilaginomycetes and Pucciniomycetes belong to Basidiomycota, the second most abundant phylum comprising some genera potentially pathogenic to maize plants, namely, *Ustilago* sp., where it includes the species *Ustilago maydis*, known for the ability to cause tumors in maize by redirecting vegetative and floral development (Walbot and Skibbe, 2009). In the class Pucciniomycetes, the genus *Puccinia* includes species responsible for the foliar disease of maize (Medina and López, 2007). Within the class Mortierellomycetes (phylum Zygomycota), the genus *Morteriella* was present in our dataset and was correlated with the organic farming system. In terms of its potential ecological role, this genus has been implicated in preventing soil degradation, improvement of soil health, and stimulating the production of plant growth hormones (Li et al., 2009; Zhang et al., 2011). Finally, the class Glomeromycete (phylum Glomeromycota) included an important functional group of organisms, the arbuscular mycorrhizae, which, according to the bibliography, can establish beneficial symbiosis with maize in various geographic distributions and different environments (Miransari et al., 2009).

Our dataset comprised 12 genera belonging to this class in the rhizosphere of both crop populations (“Pigarro” and “SinPre”) mostly associated with the organic farming system and lacking in most samples derived from the conventional farming system. Among the plant-associated mycobiota, these fungi are the most widespread (Desirò et al., 2014), and approximately 80% of the existing plant families have the potential to form this type of association (Trappe, 1987). Among the different alternatives toward sustainable agriculture, increasing arbuscular mycorrhizae is an important strategy with beneficial effects for the plant nutritional status by increasing plant nutrient uptake, in particular phosphorus (Smith and Jakobsen, 2013), improving the phytosanitary status (Ahmed et al., 2013; Hohmann and Messmer, 2017), expanding root range, improving tolerance to biotic and abiotic stresses, and inducing plant defense response (Oehl and Sieverding, 2004; Ismail et al., 2013). From our results, the organic farming system promotes these symbioses in the associated rhizosphere of these two Portuguese landraces, aiding the resilience of the crop without the insertion of synthetic fertilizers. *Dentiscutata*, *Gigaspora*, and *Paraglomus* were the most representative genera in the mycorrhizae community in our dataset and, along with *Acaulospora*, *Cetraspora*, *Diversispora*, *Glomus*, and *Scutellospora*, were restricted to the rhizosphere in the organic farming system; *Septoglomus* was genotype-specific in “SinPre.” *Glomus* belongs to the important group of arbuscular mycorrhizal (AM) fungi involved in the exchange with the plant of carbon, phosphorus, and other physiologically significant particles. As mentioned above, this interaction of mycorrhizae with other soil microorganisms promotes beneficial cooperation enhancing the competition against phytopathogenic microorganisms. Relating the rhizosphere fungal communities with their trophic mode, as expected, the abundance of taxa with saprotrophic and pathotroph modes was higher in the organic farming system than in the conventional farming system.

### Impact of Maize Populations and Farming System on the Associated Rhizosphere Bacterial Communities

There was also a clear population–farming system interaction concerning bacterial communities. A higher heterogenicity was found in the rhizosphere bacteriota under the organic farming system, in line with previous works (Hartmann et al., 2015; Lupatini et al., 2017; Fernandez et al., 2020; Lee et al., 2020; Figure 3D). Differences between populations were more pronounced in the organic farming system due to the presence of members from the classes Alcaligenaceae, Caulobacteraceae, Cellvibrionaceae, Chitinophagaceae, Intrasporangiaceae, Hyphomicrobiaceae, Opitutaceae, Planctomycetaceae, Rhodospirillaceae, and Sphingomonadaceae in the “SinPre” population, while Acidobacteriaceae, Bradyrhizobiaceae, Caldicoprobacteraceae, Comamonadaceae, Cystobacterineae, Hyphomicrobiaceae, Micromonosporaceae, Nocardioidaceae, Nocardiopsaceae, Planococcaceae, Pseudonocardiaceae, Rhizobiaceae, Rhodanobacteraceae, Rhodospirillaceae, Solirubrobacteraceae, and Steroidobacteraceae were present in “Pigarro.” These results are in line with the recent studies of Beirinckx et al. (2020), confirming the existence of a core bacteriome in the maize rhizosphere composed of Hyphomicrobiacea, Streptomycetaceae, Comamonadaceae, Cytophagaceae, Oxalobacteraceae, Rhizobacteraceae, Xanthomonadaceae, and Caulobacteraceae. Also, several taxa from these families were recently associated with the maize rhizosphere soils of various farming systems and growth stages.

The family Oxalobacteraceae was specific to the conventional farming system and has been associated with nitrogen fixation activities. However, in this study, two of the most abundant genera of this family were *Ralstonia*, a well-known plant pathogen responsible for wilting in numerous plants, and *Janthinobacterium*, previously reported in maize residues and stalks and the rhizospheric soil of maize. The importance of the latter resides in the effective reduction of maize stalk colonization by *Fusarium* spp. (Cobo-Díaz et al., 2019).

Genera from the families Xanthomonadaceae (*Luteimonas* sp.) and Comamonadaceae (M*ethylibium* sp.) were associated with the conventional farming system in our dataset, which have been previously used in the bioremediation of hydrocarbon-contaminated soil (Kane et al., 2007; Mu et al., 2016). The latter can also induce disease resistance through the production of antimicrobial compounds (Lagos et al., 2015). In line with this, it is important to highlight that we identified in our dataset families that include genera with reported biocontrol activity, such as Sphingobacteriaceae (*Pedobacter* sp.), Pseudomonadaceae (*Pseudomonas* sp.), Sphingomonadaceae (*Sphingobium* sp.), Xhantomonadaceae (*Luteibacter* sp.), Cytophagaceae (*Dyadobacter* sp.), and Rhizobiaceae (*Rhizobium* sp.) (Cobo-Díaz et al., 2019).

The main bacterial classes that contributed to differentiate the communities associated with farming systems were Bacilli and Verrucomicrobiae in the conventional farming system and Alphaproteobacteria, Anaerolineae, Fimbriimonadia, Mollicutes, Nitrospira, Opitutae, Planctomycetia, Spartobacteria, and Solibacteres in the organic farming system. Several bacterial genera of these classes were recently associated with maize rhizosphere-associated microbiota. At the genus level, *Bacillus*, *Erwinia*, *Pseudomonas*, *Stenotrophomonas*, *Achromobacter*, *Lysinibacillus*, and *Paenibacillus* were reported by Pereira et al. (2011) as the most common taxa in the maize rhizosphere. These genera, along with *Burkholderia*, were described as having plant growth-promoting rhizobacteria common in the maize-associated rhizosphere (Yang et al., 2017). Also, the antagonist potential of the genus *Bacillus* against maize pathogens has been addressed (Figueroa-López et al., 2016; Cheng et al., 2019; Hazarika et al., 2019; Liu et al., 2020). In terms of putative functions, *Burkholderia* has been linked with antifungal activity (Stopnisek et al., 2016), while some strains were described to produce ACC deaminase and siderophores important for maize growth promotion (Byrt et al., 2011). On the other hand, *Erwinia* is the causal agent of the bacterial wilt in a large range of hosts and the bacterial stalk rot disease of maize. Finally, the genus *Kaistobacter* is common in soils with atrazine (a compound present in some herbicides) (Lin et al., 2018), while *Rhodoplanes* can increase the fertility of soils (Sun et al., 2015).

## Conclusion

In the current context of climate change, environmental degradation, and misuse of natural resources, the transition from conventional to organic farming requires the optimization of cultivars and management, with the rhizosphere microbiota playing an important role. Since maize is one of the main crops in the world, the adoption of more sustainable practices will contribute decisively to this strategy. In this study, the analysis of the microbial community revealed significant differences among maize populations, showing that the plant genotype has a significant effect on the structure of the microbiota associated with the rhizosphere. Additionally, the farming system had a statistically significant impact on rhizosphere-associated microbiota, and several taxa were found to be specific to the agricultural system. The presence of arbuscular mycorrhizae (Glomeromycota), known for the potential to establish a beneficial symbiosis with maize, was mainly detected in the microbiota of the “SinPre” population in the organic farming system, being very rare under the conventional system. The diversity of the rhizosphere-associated microbiota in the organic farming system was significantly higher than that in the conventional system in both varieties, shedding some light into the potential beneficial impacts of maize landraces and organic cultivation techniques into the rhizosphere microbial communities and network assembly. The role of some species as plant growth-promoting rhizobacteria and with the ability to improve the tolerance of crops to stress conditions (biotic and abiotic) has been previously described, including in some of the genera detected in this study, namely, *Achromobacter*, *Burkholderia*, *Lysinibacillus*, *Paenibacillus*, and *Stenotrophomonas*. The role of these organisms in the sustainability and production of maize will be evaluated in future studies.

## Data Availability Statement

The datasets presented in this study can be found in online repositories. The names of the repository/repositories and accession number(s) can be found below: https://www.ncbi.nlm.nih.gov/, PRJNA675280.

## Author Contributions

AA, CJ, DP, and DS helped in the investigation. AA, JC, CJ, and DP did the formal analysis. JC and DP helped in funding acquisition, project administration, resources, and supervision. AA and JC contributed to visualization and writing of the original draft. AA, JC, MM, and DP contributed to the conceptualization, reviewed, edited, and wrote the final manuscript. All authors contributed to the article and approved the submitted version.

## Disclaimer

The opinions expressed and the arguments employed herein do not necessarily reflect the official views of the EC and the Swiss government. Neither the European Commission/SERI nor any person acting on behalf of the Commission/SERI is responsible for the use which might be made of the information provided on this website.

## Conflict of Interest

The authors declare that the research was conducted in the absence of any commercial or financial relationships that could be construed as a potential conflict of interest.

## Supplementary Material

Supplementary Figure 1 LEfSe was used to identify the most differentially abundant taxa among population genotypes in the rhizosphere of maize. Bar graph showing LDA scores for fungi **(A,B)** and bacteria **(C,D)** are represented. The length of the bar column represents the LDA score. The figure shows the microbial taxa with significant differences between the “Pigarro” (red) and “SinPre” (green). Only taxa meeting an LDA significant threshold > 2 are shown.

Supplementary Figure 2 Heat-map of the relative abundance with distance measure using Euclidean, at the level class for fungal **(A)** and bacterial **(B)** community. The relative abundance is scaled by a color gradient bar.

Supplementary Table 1 Soil characterization of the trial locations in august 2019.

Supplementary Table 2 Characterization of maize populations using HUNTERS descriptors in conventional and organic farming systems.

Supplementary Table 3 Abundance of fungal genera detected in each sample and the respective taxonomic assignment.

Supplementary Table 4 Abundance of bacterial genera detected in each sample and the respective taxonomic assignment.

Supplementary Table 5 Fungal and bacterial core rhizosphere microbiota associated with maize.

Supplementary Table 6 Fungal and bacterial genera specific to the rhizosphere microbiota in each population.

Supplementary Table 7 Fungal and bacterial genera specific to the rhizosphere microbiota of maize in distinct farming system.

Supplementary Table 8 Genera relative abundance significantly different between “Pigarro” and “SinPre” populations (*P* < 0.05).
