# Supplementary material for: Effect of Low-Input Organic and Conventional Farming Systems on Maize Rhizosphere in Two Portuguese Open-Pollinated Varieties (OPV), “Pigarro” (Improved Landrace) and “SinPre” (a Composite Cross Population)
Source: Front Microbiol. 2021 Feb 26;12:636009. doi: 10.3389/fmicb.2021.636009 (PMC7953162; doi:10.3389/fmicb.2021.636009)
Supplement: Supplementary Figure 1 — LEfSe was used to identify the most differentially abundant taxa among population genotypes in the rhizosphere of maize. Bar graph showing LDA scores for fungi (A,B) and bacteria (C,D) are represented. The length of the bar column represents the LDA score. The figure shows the microbial taxa with significant differences between the “Pigarro” (red) and “SinPre” (green). Only taxa meeting an LDA significant threshold > 2 are shown. [file Image_1.pdf]

frontiers

Supplementary Material

Effect of Low Input Organic and Conventional farming systems on maize rhizosphere in two Portuguese OPV, 'Pigarro' (improved landrace) and 'SinPre' (a Composite Cross Population)

Aitana Ares, Joana Costa*, Carolina Joaquim, Duarte Pintado, Daniela Santos, Monika M. Messmer, Pedro Mendes-Moreira

A

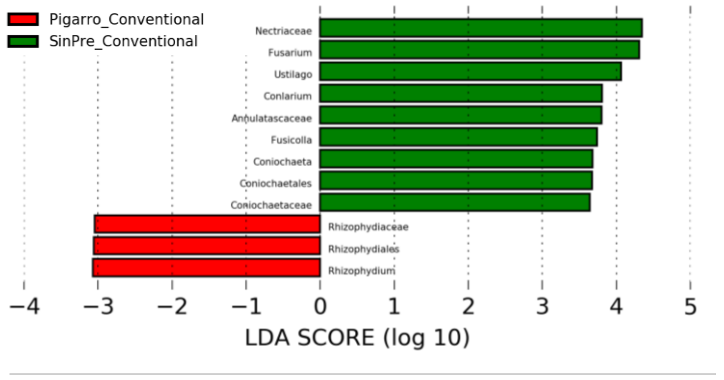

B

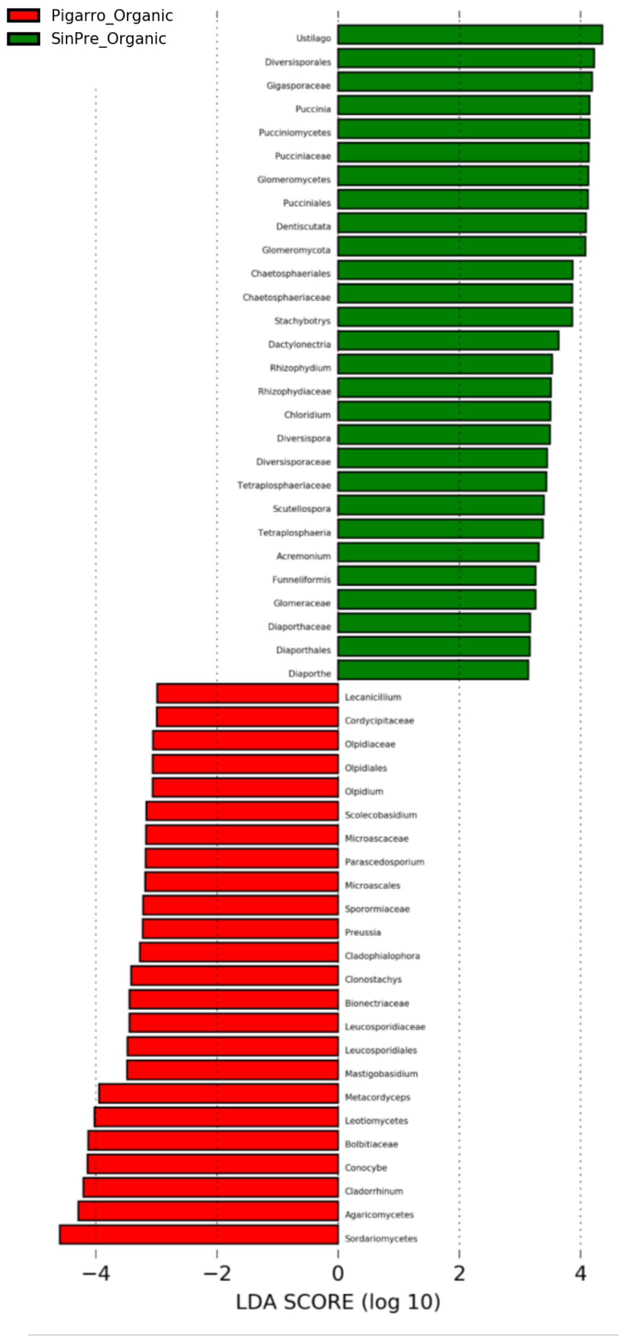

C

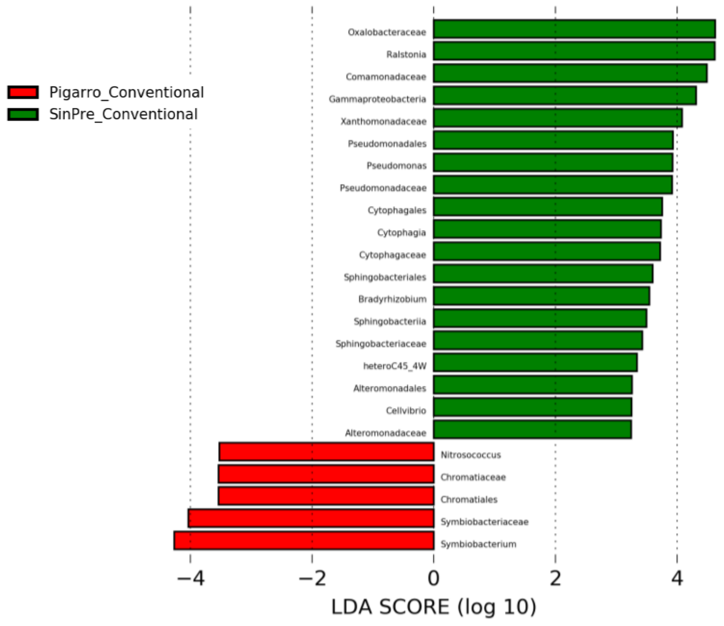

D

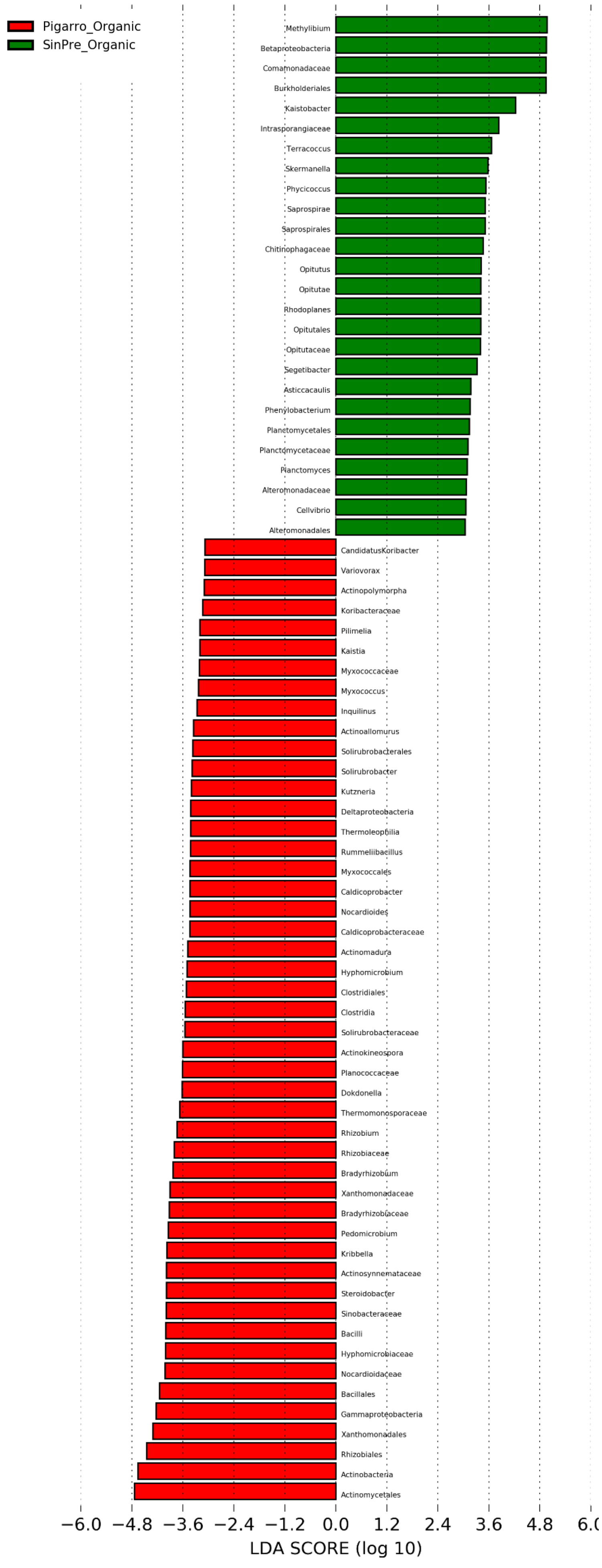

Supplementary Figure 1 | LEfSe was used to identify the most differentially abundant taxa among population genotypes in the rhizosphere of maize. Bar graph showing LDA scores for fungi (A, B) and bacteria (C, D) are represented. The length of the bar column represents the LDA score. The figure shows the microbial taxa with significant differences between the Pigarro (red) and SinPre (green). Only taxa meeting an LDA significant threshold >2 are shown.
