# Supplementary material for: Effect of Low-Input Organic and Conventional Farming Systems on Maize Rhizosphere in Two Portuguese Open-Pollinated Varieties (OPV), “Pigarro” (Improved Landrace) and “SinPre” (a Composite Cross Population)
Source: Front Microbiol. 2021 Feb 26;12:636009. doi: 10.3389/fmicb.2021.636009 (PMC7953162; doi:10.3389/fmicb.2021.636009)
Supplement: Supplementary Figure 2 — Heat-map of the relative abundance with distance measure using Euclidean, at the level class for fungal (A) and bacterial (B) community. The relative abundance is scaled by a color gradient bar. [file Image_2.pdf]

Supplementary Material

Effect of Low Input Organic and Conventional farming systems on maize rhizosphere in two Portuguese OPV, 'Pigarro' (improved landrace) and 'SinPre' (a Composite Cross Population)

Aitana Ares, Joana Costa*, Carolina Joaquim, Duarte Pintado, Daniela Santos, Monika M. Messmer, Pedro Mendes-Moreira

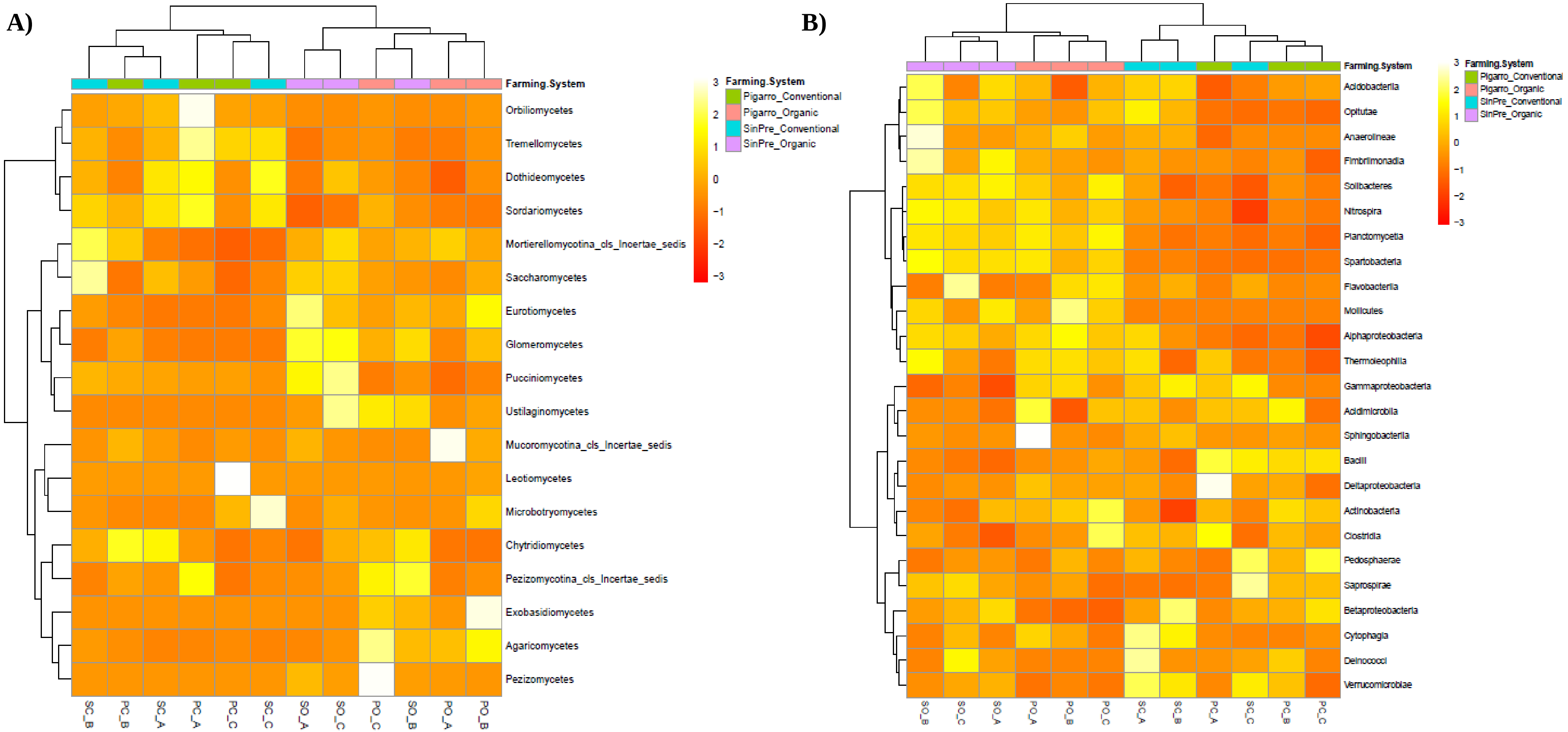

Supplementary Figure 2. Heat-map of the relative abundance with distance measure using Euclidean, at the level class for fungal (A) and bacterial (B) community. The relative abundance is scaled by a color gradient bar.
