# Supplementary material for: Effect of Low-Input Organic and Conventional Farming Systems on Maize Rhizosphere in Two Portuguese Open-Pollinated Varieties (OPV), “Pigarro” (Improved Landrace) and “SinPre” (a Composite Cross Population)
Source: Front Microbiol. 2021 Feb 26;12:636009. doi: 10.3389/fmicb.2021.636009 (PMC7953162; doi:10.3389/fmicb.2021.636009)
Supplement: Supplementary Table 1 — Soil characterization of the trial locations in august 2019. [file Table_1.pdf]

Supplementary Material

Effect of Low Input Organic and Conventional farming systems on maize rhizosphere in two Portuguese OPV, ‘Pigarro’ (improved landrace) and ‘SinPre’ (a Composite Cross Population)

Aitana Ares, Joana Costa*, Carolina Joaquim, Duarte Pintado, Daniela Santos, Monika M. Messmer, Pedro Mendes-Moreira

Supplementary Table 1. Soil characterization of the trial locations in august 2019

Soil parameter	Organic Farming System	Conventional Farming System
Field texture	Medium	Medium
Organic matter	1.8%	0.8%
pH	6.4	6.7
Available phosphorus	High	Very high
Available potassium	Very high	High
