# Supplementary material for: Effect of Low-Input Organic and Conventional Farming Systems on Maize Rhizosphere in Two Portuguese Open-Pollinated Varieties (OPV), “Pigarro” (Improved Landrace) and “SinPre” (a Composite Cross Population)
Source: Front Microbiol. 2021 Feb 26;12:636009. doi: 10.3389/fmicb.2021.636009 (PMC7953162; doi:10.3389/fmicb.2021.636009)
Supplement: Supplementary Table 2 — Characterization of maize populations using HUNTERS descriptors in conventional and organic farming systems. [file Table_2.pdf]

# Effect of Low Input Organic and Conventional farming systems on maize rhizosphere in two Portuguese OPV, 'Pigarro' (improved landrace) and 'SinPre' (a Composite Cross Population)

**Aitana Ares, Joana Costa*, Carolina Joaquim, Duarte Pintado, Daniela Santos, Monika M. Messmer, Pedro Mendes-Moreira**

**Supplementary Table 2.** Characterization of maize populations using HUNTERS descriptors in conventional and organic farming systems.

| | 'SinPre' | | 'Pigarro' | |
|---|---|---|---|---|
| | Organic | Conventional | Organic | Conventional |
| H | 206.2±24.45 | 262.3±24.86 | 250.4±22.09 | 236.4±25.46 |
| H1E | 114.6±21.12 | 159.85±22.4 | 152.6±19.47 | 174.3± 21.61 |
| U | 2 | 2.7 ±0.58 | 4 | 2 |
| N | 6 | 6 | 5 | 5.3±0.58 |
| T | 5 | 5.3±0.58 | 5 | 5.3±0.58 |
| E | 5 | 5.3±0.58 | 5.7±0.58 | 6.3 |
| %R | 0 | 0 | 2.5±0.89 | 4.1±4.81 |
| %S | 3 | 17.1±6.59 | 10.5±1.15 | 16±5.26 |

height (H); the height of 1st ear insertion (H1E), uniformity (U), Root (R%) and Stalk lodging percentage (S%).
