# Supplementary material for: Effect of Low-Input Organic and Conventional Farming Systems on Maize Rhizosphere in Two Portuguese Open-Pollinated Varieties (OPV), “Pigarro” (Improved Landrace) and “SinPre” (a Composite Cross Population)
Source: Front Microbiol. 2021 Feb 26;12:636009. doi: 10.3389/fmicb.2021.636009 (PMC7953162; doi:10.3389/fmicb.2021.636009)
Supplement: Supplementary Table 3 — Abundance of fungal genera detected in each sample and the respective taxonomic assignment. [file Table_3.pdf]

frontiers

Supplementary Material

Effect of Low Input Organic and Conventional farming systems on maize rhizosphere in two Portuguese OPV, 'Pigarro' (improved landrace) and 'SinPre' (a Composite Cross Population)

Aitana Ares, Joana Costa*, Carolina Joaquim, Duarte Pintado, Daniela Santos, Monika M. Messmer, Pedro Mendes-Moreira

* Correspondence: Corresponding Author: jcosta@uc.pt

Supplementary Table 3. Abundance of fungal genera detected in each sample and the respective taxonomic assignment.

							Abundance of genera											
**Domain**	**Phyla**	**Class**	**Order**	**Family**	**Genus**	**Nº OTUs**	**PC_A**	**PC_B**	**PC_C**	**PO_A**	**PO_B**	**PO_C**	**SC_A**	**SC_B**	**SC_C**	**SO_A**	**SO_B**	**SO_C**
		Dothideomycetes	Botryosphaeriales	Botryosphaeriaceae	*Macrophomina*	1	0	0	2	0	0	0	0	0	0	0	0	0
			Capnodiales	Davidiellaceae	*Cladosporium*	7	0	1	0	0	0	0	0	1	0	0	0	0
							0	1	0	1	0	0	0	0	0	0	0	0
							0	0	0	0	1	0	0	0	0	0	1	0
							0	0	0	2	0	0	0	0	0	0	0	0
							0	1	0	0	0	0	0	1	0	0	0	0
							0	1	0	0	0	1	0	0	0	0	0	0
							0	1	0	0	0	0	0	1	0	0	0	0
				Mycosphaerellaceae	*Ramularia*	2	0	0	0	0	0	0	0	0	0	0	2	0
							0	0	0	0	9	0	0	0	0	0	0	0
					Cercospora	2	0	1	0	0	1	0	0	0	0	0	0	0
							0	0	1	1	0	0	0	0	0	0	0	0
			Dothideales	Dothioraceae	*Pringsheimia*	1	0	2	0	0	0	0	0	0	0	0	0	0
					Aureobasidium	3	0	1	1	0	0	0	0	0	0	0	0	0
							0	1	0	2	0	0	0	0	0	0	0	0
							0	0	0	0	3	0	0	0	0	0	0	0
			Dothideomycetes_ord_Incertae_sedis	Dothideomycetes_fam_Incertae_sedis	*Cladoriella*	1	0	0	0	3	0	0	0	0	0	0	0	0
			Dothideomycetidae_ord_Incertae_sedis	Eremomycetaceae	*Arthrographis*	3	0	0	0	0	0	0	0	0	0	3	0	0
							0	0	0	1	0	0	0	0	0	2	0	0
							0	0	0	0	0	0	3	0	0	0	0	0
			Pleosporales	Lophiostomataceae	*Lophiostoma*	5	0	0	2	0	1	0	0	0	0	0	0	0
							0	1	1	0	0	0	0	1	0	0	0	0
							0	0	2	0	0	0	0	1	0	0	0	0
							0	0	0	0	0	3	0	0	0	0	0	0
							0	0	0	0	0	0	0	0	0	1	2	0
					Acrocalymma	1	0	2	0	0	0	0	1	0	0	0	0	0
				Massarinaceae	*Saccharicola*	1	0	2	2	0	0	0	0	0	0	0	0	0
				Montagnulaceae	*Paraconiothyrium*	1	0	1	1	0	0	0	0	2	0	0	0	0
				Phaeosphaeriaceae	*Stagonospora*	2	0	0	0	1	0	0	0	0	0	0	5	0
							0	0	0	0	0	0	0	0	0	0	0	7
					Setophaeosphaeria	1	0	0	0	0	0	0	0	0	0	0	7	0
					Ophiosphaerella	1	0	0	0	2	0	0	0	0	0	1	3	1
				Pleosporaceae	*Exserohilum*	1	0	0	0	8	0	0	0	0	0	0	0	0
					Drechslera	2	0	0	0	0	0	0	0	0	0	0	9	0
							0	0	0	6	1	0	0	0	0	0	2	0
					Dendryphion	1	0	0	0	9	0	0	0	0	0	0	0	0
					Curvularia	1	0	0	0	0	12	0	0	0	0	0	0	0
					Chalastospora	1	0	0	0	0	0	14	0	0	0	0	0	0
					Alternaria	13	0	5	3	0	1	0	1	3	1	0	0	0
							0	0	0	0	14	0	0	0	0	0	0	0
							0	0	0	19	0	0	0	0	0	0	0	0
							0	0	0	0	0	0	0	0	0	0	20	0
							0	0	0	0	10	3	0	0	0	0	14	0
							0	0	1	3	0	6	0	0	0	15	5	0
							0	0	0	0	31	0	0	0	0	0	0	0
							2	4	3	0	0	0	28	0	0	0	0	0
							2	20	4	0	4	0	2	6	3	0	0	0
							0	0	0	0	40	0	0	0	0	0	0	6
							1	29	3	0	2	4	2	6	6	0	0	0
							0	0	0	0	0	0	0	0	0	0	0	61
							0	0	0	47	9	9	0	0	0	0	0	0
					Pyrenochaeta	6	4	7	20	0	0	0	1	39	1	0	0	0
							2	7	0	22	4	0	38	23	5	0	3	0
							0	0	0	0	105	0	0	0	0	0	0	0

				Pleosporales_fam_Incertae_sedis	*Pyrenochaeta*	6	0	118	0	0	0	0	0	0	0	0	0	0
							0	0	0	102	1	4	0	16	0	0	2	0
							0	0	0	128	0	0	0	0	0	0	0	0
					Periconia	2	0	0	0	62	96	0	0	0	0	0	0	0
							2	0	0	39	0	0	0	0	0	172	0	18
					Berkleasmium	1	14	21	8	57	26	75	10	1	1	9	24	64
				Sporormiaceae	*Westerdykella*	3	9	6	16	0	142	23	10	3	2	119	67	16
							0	0	0	0	0	0	0	440	0	0	0	0
							11	5	9	0	149	22	6	27	1	90	66	62
					Preussia	5	0	500	0	0	0	0	0	0	0	0	0	0
							1	4	47	0	0	2	13	38	10	110	242	99
							70	21	27	55	216	15	149	28	22	31	63	30
							0	0	0	0	816	0	0	0	0	0	0	0
							8	87	320	7	0	14	102	281	45	1	1	52
				Tetraplosphaeriaceae	*Tetraplosphaeria*	1	0	0	7	671	105	184	16	1	0	6	107	3
			Tubeufiales	Tubeufiaceae	*Helicoma*	1	12	198	617	3	2	0	43	166	107	0	0	0
			Venturiales	Venturiaceae	*Veronaeopsis*	4	2	21	22	612	166	150	17	14	11	195	199	158
							4	28	580	4	1139	0	0	30	12	121	99	0
							11	0	41	935	104	341	0	0	0	610	97	114
							122	445	172	327	47	59	224	174	80	168	276	229
			Arachnomycetales	Arachnomycetaceae	*Arachnomyces*	3	119	269	43	828	218	239	61	53	36	388	132	182
							5	0	66	800	456	276	0	0	0	909	1295	319
							72	212	92	367	455	138	137	165	59	423	1322	809
			Chaetothyriales	Chaetothyriaceae	*Cyphellophora*	2	36	2243	571	0	139	149	144	799	334	116	76	40
							0	0	484	3385	493	378	1	31	0	3052	492	506
				Herpotrichiellaceae	*Exophiala*	25	65	3607	981	18737	128	108	536	65	257	559	113	39
							0	0	0	0	0	0	0	0	0	2	0	0
							0	0	0	2	0	0	0	0	0	0	0	0
							0	0	0	0	0	0	0	0	0	1	1	0
							0	0	0	2	0	0	0	0	0	0	0	0
							0	0	0	0	0	0	0	0	0	0	2	0
							0	0	0	0	0	0	0	0	0	0	2	0
							0	0	0	0	0	0	0	0	0	0	2	0
							0	0	0	0	0	0	0	0	0	0	2	0
							0	0	0	0	2	0	0	0	0	0	0	0
							0	1	2	0	0	0	0	0	0	0	0	0
							0	0	0	0	0	0	0	3	0	0	0	0
							0	0	0	0	0	2	1	0	0	0	0	0
							0	0	0	0	0	0	0	0	0	0	3	0
							0	0	0	0	2	0	0	0	0	1	0	0
							0	0	0	0	0	0	0	0	0	3	0	0
							0	0	0	0	0	2	0	0	0	0	2	0
							0	0	0	0	0	0	0	0	0	0	4	0
							0	0	0	0	0	0	0	0	0	4	0	0
							0	0	0	0	0	0	0	0	0	3	0	1
							0	0	0	0	2	0	0	0	0	0	2	0
							0	0	0	0	0	4	0	0	0	0	0	0
							0	0	0	0	0	0	5	0	0	0	0	0
							0	0	0	0	1	0	0	0	0	1	0	3
							0	0	6	0	0	0	0	0	0	0	0	0
					Cladophialophora	5	0	0	0	0	0	0	0	0	0	0	6	0
							0	0	0	0	0	0	0	0	0	1	3	3
							0	0	0	0	0	5	0	0	0	0	2	0
							0	0	0	3	0	0	0	0	0	0	1	3
							0	0	0	0	0	0	0	0	0	0	0	7
				Eurotiales_fam_Incertae_sedis	*Thermomyces*	3	0	0	0	2	0	0	0	0	0	0	0	5
							0	0	0	0	2	0	0	0	0	0	5	0
							0	0	0	0	0	0	0	0	0	5	1	1
					Leiothecium	1	0	0	0	0	0	0	0	0	0	0	7	1
					Talaromyces	22	0	0	0	0	0	0	0	0	0	8	0	0
							0	0	0	1	0	0	0	0	0	0	7	0
							0	0	0	0	0	0	2	6	0	0	0	0
							0	0	0	0	0	0	0	0	0	2	6	0
							0	0	0	0	1	0	0	0	0	1	7	0
							0	3	0	0	0	0	0	0	0	3	0	3
							0	0	0	1	0	0	0	0	0	0	0	8
							0	0	0	5	1	2	0	0	0	0	2	0
							0	0	0	0	0	1	0	0	0	0	7	2
							0	0	0	0	0	0	0	0	0	0	9	1
							0	0	0	0	1	1	0	0	0	0	11	1
							0	0	0	0	6	0	0	0	0	6	2	0

							0	0	0	0	0	0	0	0	0	2	4	11
							0	0	0	2	0	1	0	0	0	0	15	0
							0	0	0	0	0	6	0	0	0	12	0	0
							0	0	0	11	0	2	0	0	0	0	7	0
							0	0	0	0	0	0	0	20	0	0	0	0
							0	0	0	0	0	0	0	0	0	25	0	0
							0	0	0	0	0	2	0	0	0	23	0	0
							0	0	0	9	0	3	0	0	0	1	20	0
							0	0	0	10	0	4	0	0	0	9	8	7
							0	0	0	0	19	0	0	0	0	9	11	0
							0	0	0	0	0	0	0	0	0	43	0	0
							0	23	0	0	2	2	1	0	0	16	0	6
							0	3	0	0	0	5	0	0	0	4	35	7
							0	2	0	3	1	11	0	0	0	15	16	6
							0	3	0	3	4	9	0	0	0	22	20	8
							0	0	0	21	4	13	0	0	0	14	18	2
							0	12	0	8	0	2	0	0	0	5	8	37
							0	0	0	5	27	2	0	0	0	4	41	0
							0	39	0	2	7	0	1	0	0	32	0	5
							0	2	0	0	0	10	0	0	0	21	51	4
							0	37	0	0	5	3	0	0	0	37	0	11
							0	0	0	7	1	3	0	0	0	46	12	29
							0	0	0	4	0	12	0	0	0	32	34	16
							0	1	0	0	4	19	0	0	0	37	29	17
							0	24	2	40	1	2	3	0	0	21	17	17
							0	0	0	0	137	0	0	0	0	0	0	0
							0	45	0	3	4	14	2	0	0	37	20	15
							0	0	0	11	11	1	0	0	0	62	54	12
							0	86	0	6	29	1	3	0	0	43	6	18
							0	1	0	7	10	17	3	2	0	95	66	19
							0	0	0	4	0	0	0	0	0	90	58	94
							0	0	0	8	16	20	0	0	0	113	79	53
							0	126	0	8	17	6	5	3	0	112	0	25
							0	225	0	22	54	2	5	0	0	170	24	39
							0	0	0	24	0	4	0	0	0	263	185	342
							0	0	0	70	364	139	0	0	0	115	393	139
							0	0	0	5	220	297	0	0	0	1085	935	377
							0	0	0	0	0	0	0	0	0	1	1	0
							0	0	0	0	2	0	0	0	0	0	0	0
							2	0	0	0	0	0	0	0	0	0	0	0
					Penicillium	62	0	0	0	0	0	0	0	0	2	0	0	0
							0	0	0	0	0	0	0	0	0	0	2	0
							0	0	0	0	0	0	0	0	0	0	0	2
							0	0	0	0	0	0	0	2	0	0	0	0
							0	0	0	0	0	0	0	0	0	0	0	3
		Eurotiomycetes					0	0	0	0	0	0	2	1	0	0	0	0
							0	0	0	0	0	0	3	1	0	0	0	0
							0	0	3	0	0	0	0	0	1	0	0	0
							0	0	0	8	0	0	0	0	0	0	0	0
							0	1	0	1	0	5	0	0	1	0	0	0
							4	0	7	0	0	0	0	0	0	0	0	0
							0	0	0	0	0	0	0	0	0	0	0	12
							0	0	0	0	0	10	0	0	3	0	0	0
							7	1	0	0	0	0	1	5	0	0	0	0
			Eurotiales				0	0	0	17	0	0	0	0	0	0	1	0
							0	0	0	0	0	0	0	0	36	0	0	0
				Trichocomaceae			0	0	0	0	28	17	0	0	0	0	4	0
							9	1	6	0	0	0	0	0	0	6	29	7
							0	71	0	0	0	0	0	0	0	0	0	0
							15	0	5	0	0	0	0	0	0	41	21	0
							0	0	0	7	0	44	0	0	0	53	0	6
							8	0	0	0	0	0	0	0	0	115	0	0
							0	0	0	0	0	0	0	0	0	0	182	0
							0	0	0	0	0	0	185	32	2	0	0	0
							0	0	0	0	0	0	0	0	219	0	0	0
							4	11	24	21	0	0	9	0	333	0	56	2
							0	0	0	0	580	0	0	0	0	0	0	0
							93	1304	0	0	0	0	0	0	17	0	0	0
							346	567	35	0	0	0	296	35	41	0	253	2
							18	150	10	6	0	24	27	183	54	2	9	1973
							87	1011	45	20	23	1	2886	805	93	0	0	0

							1	5700	3	176	1	0	2	0	0	38	273	6
					Paecilomyces	4	0	0	0	8	13	3112	0	0	0	4	8021	6
							0	0	0	0	0	0	0	0	2	0	0	0
							0	0	0	0	0	2	0	0	0	0	0	0
							0	0	0	0	0	0	0	1	1	0	0	0
					Aspergillus	49	0	0	0	0	0	0	0	0	0	0	2	0
							2	0	0	0	0	0	0	0	0	0	0	0
							0	0	0	0	0	0	0	0	0	0	2	0
							0	2	0	0	0	0	0	0	0	0	0	0
							0	0	0	0	0	0	0	0	0	0	0	2
							0	0	0	0	2	0	0	0	0	0	0	0
							0	0	0	0	0	0	0	0	0	0	2	0
							0	0	2	0	0	0	0	0	0	0	0	0
							0	0	0	0	0	0	0	0	0	2	0	0
							0	0	0	0	0	2	0	0	0	0	0	0
							0	0	0	0	0	0	0	1	0	1	0	0
							0	0	0	0	0	0	1	0	0	1	0	0
							0	0	0	0	0	0	0	2	0	0	0	0
							0	0	0	0	0	2	0	0	0	0	0	0
							0	0	0	0	0	0	0	0	3	0	0	0
							0	0	0	0	0	0	3	0	0	0	0	0
							0	0	0	0	0	0	0	0	0	3	0	0
							1	0	0	0	2	0	0	0	0	0	0	0
							0	1	0	0	2	0	0	0	0	0	0	0
							0	0	0	0	0	0	0	0	0	0	3	0
							0	0	0	0	0	0	0	0	0	3	0	0
							0	0	0	2	0	0	0	0	0	0	0	1
							0	0	0	2	1	0	0	0	0	0	0	0
							0	0	0	0	1	2	0	0	0	0	0	0
							0	0	0	0	0	0	0	0	0	0	0	3
							0	0	0	0	0	3	0	0	0	0	0	0
							0	0	0	3	0	0	0	0	0	0	0	0
							0	0	0	0	0	0	0	0	0	0	0	3
							0	0	0	0	1	0	1	0	0	1	0	0
							0	0	0	0	0	1	0	0	0	2	0	0
							0	0	0	0	0	0	0	0	0	0	3	0
							0	4	0	0	0	0	0	0	0	0	0	0
							0	0	0	4	0	0	0	0	0	0	0	0
							1	0	0	0	3	0	0	0	0	0	0	0
							0	0	0	0	0	0	0	0	0	0	4	0
							0	0	0	0	0	0	0	0	0	0	0	4
							0	0	0	4	0	0	0	0	0	0	0	0
							0	0	0	0	2	0	0	0	0	0	2	0
							0	0	0	0	1	0	0	0	3	0	0	0
							0	0	0	0	0	0	0	0	0	0	4	0
							0	0	0	4	0	0	0	0	0	0	0	0
							0	4	0	0	0	0	0	0	0	0	0	0
							0	0	0	0	1	3	0	0	0	0	0	0
							0	0	0	0	0	0	0	0	0	0	0	4
							0	0	0	4	0	0	0	0	0	0	0	0
							0	0	0	0	0	2	0	0	0	0	1	1
							0	4	0	0	0	0	0	0	0	0	0	0
							0	0	0	0	0	3	0	0	0	0	1	0
							0	1	1	0	0	0	3	0	0	0	0	0
			Onygenales	Ajellomycetaceae	*Spiromastix*	4	0	0	0	0	5	0	0	0	0	0	0	0
							0	2	0	0	0	0	0	0	0	2	0	1
							0	0	0	0	0	0	0	0	0	0	0	5
							0	0	0	0	0	0	0	0	0	0	5	0
					Emmonsia	1	1	0	0	0	0	0	4	0	0	0	0	0
				Arthrodermataceae	*Trichophyton*	1	0	0	0	0	5	0	0	0	0	0	0	0
				Gymnoascaceae	*Gymnoascus*	2	0	0	0	0	0	0	0	0	0	0	0	5
							0	0	0	1	2	0	0	0	0	0	0	2
				Onygenaceae	*Chrysosporium*	6	0	2	0	0	0	0	0	0	0	0	3	0
							0	0	0	0	0	0	0	4	0	2	0	0
							0	0	0	0	6	0	0	0	0	0	0	0
							0	0	0	0	0	0	0	0	0	0	0	6
							0	0	0	0	0	6	0	0	0	0	0	0
							0	0	0	0	0	0	0	0	0	0	7	0
					Auxarthron	3	0	0	0	0	0	0	0	3	0	0	1	3
							0	0	0	0	7	0	0	0	0	0	0	0
							0	0	0	0	0	3	0	0	0	0	4	0

	Ascomycota		Verrucariales	Verrucariaceae	*Verrucaria*	1	7	0	0	0	0	0	1	0	0	0	0	0
		Leotiomycetes	Helotiales	Helotiaceae	*Neobulgaria*	1	0	0	0	0	0	0	0	0	0	0	5	3
					Articulospora	2	0	0	0	0	0	0	0	0	0	0	0	8
							0	0	0	0	1	0	0	0	0	0	7	0
				Helotiales_fam_Incertae_sedis	*Tetracladium*	2	0	0	0	0	0	8	0	0	0	0	0	0
							0	0	0	2	5	1	0	0	0	0	0	0
					Scytalidium	4	0	0	0	0	0	0	0	0	0	9	0	0
							0	5	1	0	0	0	3	0	0	0	0	0
							0	0	0	0	2	0	0	0	0	0	0	7
							0	0	0	2	0	0	0	0	0	7	0	0
					Dactylaria	3	0	0	0	0	0	0	0	0	0	8	2	0
							0	0	0	0	11	0	0	0	0	0	0	0
							0	0	0	0	5	6	0	0	0	0	0	0
				Sclerotiniaceae	*Sclerotinia*	1	0	2	0	2	1	0	7	0	0	0	0	0
					Botrytis	1	0	12	0	0	0	0	0	0	0	0	0	0
				Vibrisseaceae	*Phialocephala*	1	0	1	0	11	0	0	0	0	0	0	0	0
			Leotiomycetes_ord_Incertae_sedis	Leotiomycetes_fam_Incertae_sedis	*Geomyces*	1	0	0	0	0	12	0	0	0	0	0	0	0
				Myxotrichaceae	*Oidiodendron*	12	0	13	0	0	0	0	0	0	0	0	0	0
							1	0	1	3	4	0	0	0	0	0	0	4
							1	0	9	3	0	0	0	0	0	0	0	0
							0	0	0	0	3	0	0	0	0	10	1	0
							0	0	0	0	0	11	0	0	0	0	3	0
							0	0	0	0	9	0	0	0	0	0	5	0
							0	0	0	0	0	0	0	0	0	0	0	14
							0	0	0	0	0	0	15	0	0	0	0	0
							0	0	16	0	0	0	0	0	0	0	0	0
							0	0	0	0	0	2	0	0	0	1	0	14
							0	0	0	0	0	0	0	0	0	17	0	0
							0	0	0	0	0	0	0	0	0	0	17	0
					Malbranchea	1	0	0	0	4	0	0	8	6	0	0	0	0
		Orbiliomycetes	Orbiliales	Orbiliaceae	*Brachyphoris*	1	0	0	4	8	0	0	3	0	0	0	2	2
					Arthrobotrys	3	0	0	0	0	0	0	0	0	0	0	19	0
							0	0	1	0	0	0	3	0	15	0	0	0
							2	4	7	0	0	0	0	0	7	0	0	0
		Pezizomycetes	Pezizales	Ascobolaceae	*Ascobolus*	1	0	0	0	1	4	4	1	1	0	2	0	8
				Pezizaceae	*Peziza*	2	0	0	0	0	21	0	0	0	0	0	0	0
							0	0	0	0	0	0	0	0	0	22	0	0
				Pyronemataceae	*Scutellinia*	3	0	0	0	0	18	0	0	4	0	0	0	0
							0	0	0	0	0	23	0	0	0	0	0	0
							0	0	0	0	3	0	0	0	2	0	18	0
		Pezizomycotina_cls_Incertae_sedis	Pezizomycotina_ord_Incertae_sedis	Pezizomycotina_fam_Incertae_sedis	*Vermispora*	1	0	0	12	8	0	0	0	0	0	0	4	0
					Torula	3	0	0	0	0	24	0	0	0	0	0	0	0
							0	5	8	0	0	1	0	0	2	0	0	9
							0	0	0	0	2	25	0	0	0	0	0	0
					Staphylotrichum	4	0	0	0	0	10	0	0	0	1	16	0	0
							0	0	0	0	10	17	0	0	0	0	0	1
							0	0	12	0	0	0	0	0	0	0	0	16
							0	0	0	0	0	0	0	0	0	29	0	0
					Scolecobasidium	1	0	0	0	6	2	0	0	0	0	6	1	15
					Remersonia	2	0	0	0	0	0	0	0	0	0	27	0	4
							2	0	0	0	0	0	11	13	8	0	0	0
					Polyschema	1	0	0	0	0	0	0	0	0	0	37	0	0
					Ochroconis	2	5	4	3	0	14	0	7	0	6	0	3	0
							0	0	0	2	46	0	0	0	0	0	2	0
					Leptodiscella	1	0	6	0	7	0	0	0	0	1	28	10	0
					Clohesyomyces	3	0	0	0	0	0	0	0	0	0	56	0	0
							0	0	0	0	68	0	0	0	0	0	0	0
							0	0	0	0	76	0	0	0	0	0	0	0
					Calcarisporiella	1	0	0	0	0	0	76	0	0	0	0	0	0
		Saccharomycetes	Saccharomycetales	Lipomycetaceae	*Lipomyces*	3	0	79	3	0	0	0	0	0	1	0	0	0
							2	2	63	0	2	0	8	0	7	0	0	0
							5	13	8	5	9	0	8	4	1	18	15	2
				Pichiaceae	*Pichia*	2	1	2	40	0	0	0	1	0	44	0	0	0
							0	0	0	0	89	0	0	0	0	0	0	0
				Saccharomycetaceae	*Torulaspora*	1	0	0	0	0	25	17	0	0	0	3	5	39
					Saccharomyces	1	27	23	19	7	4	0	3	4	5	0	0	2
					Lachancea	1	0	0	0	0	0	0	0	0	0	0	106	0
					Wickerhamomyces	1	2	16	22	10	6	6	0	13	4	2	8	21
					Kodamaea	1	0	0	0	5	33	22	0	0	0	2	18	37
					Debaryomyces	1	13	1	111	0	0	0	0	0	3	0	0	0
							0	0	0	94	30	0	0	0	0	0	5	0

				Saccharomycetales_fam_Incertae_sedis	*Candida*	4	0	0	0	109	5	0	0	0	0	0	0	27
							0	0	0	5	108	36	0	0	0	0	30	0
							0	0	0	2	0	0	0	0	0	190	2	0
					Barnettozyma	2	0	0	0	4	3	155	0	0	2	36	46	5
							2	7	18	0	0	223	0	6	3	0	1	0
				Saccharomycodaceae	*Hanseniaspora*	3	1	6	6	19	88	24	0	20	1	18	11	71
							0	0	0	56	69	27	0	0	28	0	68	19
							5	0	0	0	0	0	0	0	354	0	0	0
				Saccharomycopsidaceae	*Saccharomycopsis*	1	0	0	0	365	0	0	0	0	0	0	0	0
			Calosphaeriales	Pleurostomataceae	*Pleurostoma*	1	0	0	0	0	0	8	0	0	0	0	416	8
			Chaetosphaeriales	Chaetosphaeriaceae	*Chloridium*	4	1	11	3	107	184	80	0	0	1	15	47	11
							2	4	2	54	191	35	2	0	7	25	52	103
							0	0	0	0	0	584	0	0	0	0	3	0
							6	0	3	159	533	27	0	0	17	54	127	61
					Chaetosphaeria	5	1	0	1	41	107	801	2	0	0	7	20	185
							3	55	90	0	0	792	0	42	0	0	272	2
							4	5	8	186	577	123	4	6	7	93	183	359
							0	0	0	756	777	162	0	0	0	0	19	39
							0	0	1	50	127	135	1	2	1	307	1292	1148
			Coniochaetales	Coniochaetaceae	*Coniochaeta*	9	555	278	1382	24	8	4	325	197	454	15	60	2
							104	386	511	93	145	106	186	122	150	847	379	659
							1	49	1623	1	0	0	5	115	2039	0	0	0
							0	0	0	2	0	0	0	0	0	0	0	0
							0	0	0	0	1	0	0	0	0	0	0	1
							0	0	0	0	0	0	0	0	0	2	0	0
							0	0	0	0	0	0	0	0	0	0	2	0
							0	0	0	0	1	0	0	0	0	0	1	0
							0	0	0	2	0	0	0	0	0	0	0	0
			Diaporthales	Diaporthaceae	*Diaporthe*	1	0	0	0	0	0	2	0	0	0	0	0	0
				Bionectriaceae	*Selinia*	1	0	0	0	0	0	0	0	0	0	0	0	2
					Clonostachys	6	0	0	0	0	0	2	0	0	0	0	0	0
							0	0	0	0	0	0	0	0	0	0	2	0
							0	0	0	0	0	0	0	0	0	0	2	0
							0	0	0	0	0	0	0	1	0	1	0	0
							1	1	0	0	0	0	0	0	0	0	0	0
							0	0	0	0	0	0	0	0	0	0	2	0
				Clavicipitaceae	*Metarhizium*	6	0	0	0	0	0	0	0	0	0	0	2	0
							0	0	2	0	0	0	0	0	0	0	0	0
							1	1	0	0	0	0	0	0	0	0	0	0
							0	0	0	0	0	2	0	0	0	0	0	0
							0	0	0	2	0	0	0	0	0	0	0	0
							0	0	0	1	0	0	1	0	0	0	0	0
					Metacordyceps	5	0	0	0	0	2	0	0	0	0	0	0	0
							0	0	0	0	0	0	0	0	0	0	2	0
							0	0	0	0	0	0	2	0	0	0	0	0
							0	0	0	1	0	0	0	0	0	0	1	0
							0	0	0	2	0	0	0	0	0	0	0	0
				Cordycipitaceae	*Lecanicillium*	3	0	0	0	0	0	0	0	0	2	0	0	0
							0	0	0	0	2	0	0	0	0	0	0	0
							1	0	0	0	0	0	0	1	0	0	0	0
					Beauveria	3	0	0	0	0	0	0	0	0	0	0	2	0
							0	0	0	0	0	0	1	1	0	0	0	0
							0	0	0	0	2	0	0	0	0	0	0	0
				Hypocreaceae	*Trichoderma*	22	1	0	1	0	0	0	0	0	0	0	0	0
							0	0	0	1	1	0	0	0	0	0	0	0
							0	0	0	2	0	0	0	0	0	0	0	0
							0	0	0	0	0	0	0	0	0	0	2	0
							0	0	0	0	0	1	0	0	0	0	1	0
							0	0	0	0	0	2	0	0	0	0	0	0
							0	0	1	0	0	0	0	0	0	0	1	0
							0	0	1	0	0	0	0	0	0	0	1	0
							0	0	0	2	0	0	0	0	0	0	0	0
							0	0	0	2	0	0	0	0	0	0	0	0
							0	0	0	0	2	0	0	0	0	0	0	0
							0	0	2	0	0	0	0	0	0	0	0	0
							0	0	0	1	0	0	0	0	0	1	0	0
							0	0	0	0	0	0	0	0	0	2	0	0
							0	0	0	0	0	0	0	0	0	2	0	0
							0	0	1	0	0	0	0	0	1	0	0	0
							0	0	0	0	0	0	0	0	2	0	0	0
							0	0	2	0	0	0	0	0	0	0	0	0

Sordariomycetes	Hypocreales				0	0	1	0	0	0	0	0	0	0	1	0
					0	0	1	0	0	0	0	0	1	0	0	0
					0	0	0	1	0	0	0	0	0	0	0	1
					0	0	0	0	0	0	0	0	0	0	2	0
			Acrostalagmus	1	0	0	2	0	0	0	0	0	0	0	0	0
		Hypocreales_fam_Incertae_sedis	*Stachybotrys*	6	0	0	0	0	0	0	0	0	0	2	0	0
					0	0	0	0	0	0	0	0	0	0	2	0
					0	0	0	0	0	0	0	0	0	2	0	0
					0	0	0	0	0	2	0	0	0	0	0	0
					0	0	0	0	0	0	0	0	1	1	0	0
					0	0	0	1	1	0	0	0	0	0	0	0
			Sarocladium	4	0	0	0	1	0	1	0	0	0	0	0	0
					0	0	2	0	0	0	0	0	0	0	0	0
					0	0	0	1	0	0	0	0	0	1	0	0
					0	0	0	2	0	0	0	0	0	0	0	0
			Myrothecium	3	1	0	1	0	0	0	0	0	0	0	0	0
					0	0	0	0	0	0	0	0	0	2	0	0
					0	0	2	0	0	0	0	0	0	0	0	0
			Ilyonectria	2	0	0	0	0	0	0	0	0	0	1	1	0
					0	0	0	0	0	0	0	0	0	0	2	0
			Gliomastix	1	0	0	0	0	0	0	0	0	0	0	1	1
			Acremonium	18	0	0	0	1	0	0	0	0	0	0	0	1
					0	0	0	1	0	0	0	0	0	1	0	0
					0	0	0	0	0	0	0	0	0	0	2	0
					0	0	0	0	0	0	0	0	0	2	0	0
					0	0	0	0	1	0	0	0	0	1	0	0
					0	0	0	0	0	0	0	0	0	0	2	0
					0	0	0	0	0	0	0	0	0	2	0	0
					2	0	0	0	0	0	0	0	0	0	0	0
					0	0	2	0	0	0	0	0	0	0	0	0
					2	0	0	0	0	0	0	0	0	0	0	0
					0	1	1	0	0	0	0	0	0	0	0	0
					0	0	0	1	0	0	0	0	0	0	1	0
					0	0	2	0	0	0	0	0	0	0	0	0
					0	0	0	0	0	0	0	0	0	0	2	0
					2	0	0	0	0	0	0	0	0	0	0	0
					0	0	0	0	0	0	0	0	1	0	1	0
					0	0	2	0	0	0	0	0	0	0	0	0
					2	0	0	0	0	0	0	0	0	0	0	0
		Nectriaceae	*Volutella*	1	0	0	0	0	0	0	0	0	0	2	0	0
			Stylonectria	2	0	0	0	2	0	0	0	0	0	0	0	0
					0	0	0	2	0	0	0	0	0	0	0	0
			Rectifusarium	1	0	2	0	0	0	0	0	0	0	0	0	0
			Mariannaea	2	0	0	0	0	1	0	0	0	0	0	0	1
					0	0	2	0	0	0	0	0	0	0	0	0
			Gibberella	4	0	0	0	0	0	0	0	0	0	2	0	0
					0	0	0	1	0	0	0	0	0	0	1	0
					0	0	2	0	0	0	0	0	0	0	0	0
					1	0	0	1	0	0	0	0	0	0	0	0
			Fusicolla	1	1	0	0	0	0	0	0	0	0	0	1	0
			Fusarium	42	0	0	0	0	2	0	0	0	0	0	0	0
					0	0	2	0	0	0	0	0	0	0	0	0
					0	2	0	0	0	0	0	0	0	0	0	0
					0	0	0	0	0	2	0	0	0	0	0	0
					0	0	0	2	0	0	0	0	0	0	0	0
					0	2	0	0	0	0	0	0	0	0	0	0
					0	0	0	0	0	0	0	0	0	1	0	1
					0	0	0	0	0	0	0	0	0	0	2	0
					0	0	0	0	0	0	0	0	0	1	1	0
					0	0	0	0	0	0	0	0	0	2	0	0
					0	1	0	0	1	0	0	0	0	0	0	0
					0	0	0	2	0	0	0	0	0	0	0	0
					0	0	1	0	0	0	0	0	0	1	0	0
					0	0	0	0	0	1	0	0	0	0	0	1
					0	0	0	0	1	0	0	0	0	0	1	0
					0	0	2	0	0	0	0	0	0	0	0	0
					0	0	0	1	1	0	0	0	0	0	0	0
					0	0	0	0	2	0	0	0	0	0	0	0
					1	0	0	0	0	0	0	1	0	0	0	0
					0	0	0	2	0	0	0	0	0	0	0	0
					0	0	0	0	0	0	0	0	0	0	2	0

Fungi					*Fusarium*	42	0	0	0	0	0	0	0	0	0	0	2	0
							0	0	1	0	1	0	0	1	0	0	0	0
							0	0	0	0	0	3	0	0	0	0	0	0
							0	0	0	0	0	0	0	0	0	0	0	3
							0	0	1	0	0	0	0	2	0	0	0	0
							0	0	1	0	0	1	0	1	0	0	0	0
							1	0	0	0	0	1	0	0	0	0	1	0
							0	0	0	0	0	0	0	3	0	0	0	0
							0	2	0	0	0	0	0	0	1	0	0	0
							0	0	0	0	1	0	0	0	0	2	0	0
							0	0	0	0	0	1	0	0	0	0	2	0
							0	0	2	0	0	0	0	1	0	0	0	0
							0	0	0	0	2	0	0	0	0	0	1	0
							0	0	0	0	0	0	0	0	0	0	0	3
							0	0	0	0	3	0	0	0	0	0	0	0
							0	0	0	1	0	0	0	0	0	0	2	0
							0	0	0	0	0	0	0	0	0	0	3	0
							0	0	0	0	0	0	0	0	0	0	3	0
							0	0	0	1	0	0	0	0	0	1	1	0
							0	0	0	2	1	0	0	0	0	0	0	0
							0	0	0	0	0	0	0	0	0	0	0	3
					Dactylonectria	1	0	0	0	0	3	0	0	0	0	0	0	0
					Cosmospora	1	0	3	0	0	0	0	0	0	0	0	0	0
				Ophiocordycipitaceae	*Purpureocillium*	3	0	0	0	3	0	0	0	0	0	0	0	0
							1	0	1	0	1	0	0	0	0	0	0	0
							0	0	0	0	0	0	0	0	0	0	3	0
			Hypocreomycetidae_ord_Incertae_sedis	Plectosphaerellaceae	*Lectera*	1	0	0	0	0	0	0	0	0	0	0	3	0
			Magnaporthales	Magnaporthaceae	*Buergenerula*	1	0	0	0	1	0	1	0	0	0	0	1	0
				Pyriculariaceae	*Pseudopyricularia*	1	0	0	0	0	0	2	0	0	0	1	0	0
			Microascales	Microascaceae	*Scopulariopsis*	2	0	0	0	0	0	0	0	0	0	3	0	0
							0	0	0	2	0	0	0	1	0	0	0	0
					Scedosporium	3	1	0	0	0	0	0	0	0	0	2	0	0
							0	0	0	0	2	1	0	0	0	0	0	0
							0	0	0	0	2	1	0	0	0	0	0	0
					Pithoascus	2	0	0	1	0	0	0	0	1	0	0	1	0
							0	0	2	0	0	0	1	0	0	0	0	0
					Petriella	1	0	0	0	0	0	0	0	0	0	0	3	0
					Parascedosporium	1	0	1	0	0	1	0	0	0	0	1	0	0
					Microascus	5	0	0	0	0	0	0	0	0	0	0	3	0
							0	0	0	0	0	0	0	0	0	0	3	0
							0	0	0	0	0	0	0	0	3	0	0	0
							0	0	0	1	0	0	0	0	0	0	0	2
							0	0	0	0	0	0	0	0	0	0	3	0
			Ophiostomatales	Ophiostomataceae	*Sporothrix*	3	0	0	0	0	0	0	0	0	0	3	0	0
							0	0	0	0	0	0	0	0	0	0	2	1
							0	0	0	0	3	0	0	0	0	0	0	0
				Chaetomiaceae	*Thielavia*	4	0	0	2	0	0	0	0	0	1	0	0	0
							0	0	0	1	0	0	0	0	0	0	2	0
							0	0	2	0	0	0	0	0	0	0	1	0
							0	0	0	1	0	0	0	0	0	0	0	2
					Chaetomium	19	0	0	0	0	3	0	0	0	0	0	0	0
							0	0	0	0	0	0	0	0	0	0	0	3
							0	0	0	0	0	0	0	0	0	1	2	0
							0	0	3	0	0	0	0	0	0	0	0	0
							0	0	0	0	0	0	0	0	0	3	0	0
							0	0	0	3	0	0	0	0	0	0	0	0
							0	1	0	0	0	0	0	0	0	1	1	0
							0	0	0	0	0	0	0	0	0	3	0	0
							0	0	0	2	0	0	0	0	0	0	1	0
							0	0	0	0	0	0	0	0	0	0	0	3
							0	0	3	0	0	0	0	0	0	0	0	0
							0	0	0	3	0	0	0	0	0	0	0	0
							0	1	1	0	0	1	0	0	0	0	0	0
							0	0	0	0	0	0	0	0	0	0	0	3
							0	0	0	0	0	0	0	0	0	3	0	0
							0	0	0	0	0	1	0	0	0	2	0	0
							0	0	0	0	4	0	0	0	0	0	0	0
							0	0	0	0	1	0	1	0	0	0	0	2
							0	0	0	0	0	0	0	0	0	4	0	0
					Zopfiella	4	0	0	0	0	0	0	0	0	0	4	0	0
							0	0	0	0	0	0	0	0	0	0	0	4

			Sordariales	Lasiosphaeriaceae	*Zopfiella*	4	0	0	0	0	0	4	0	0	0	0	0	0
							0	1	0	0	0	0	0	1	1	0	0	1
					Schizothecium	1	0	0	0	4	0	0	0	0	0	0	0	0
					Podospora	16	0	0	0	0	0	0	0	0	0	0	0	4
							0	0	0	4	0	0	0	0	0	0	0	0
							0	0	0	0	0	0	0	0	0	0	4	0
							0	0	0	0	0	0	0	0	0	1	3	0
							0	0	0	2	0	0	0	0	0	0	2	0
							0	0	0	0	0	0	0	1	1	0	1	1
							0	0	0	0	0	0	0	0	0	4	0	0
							0	1	0	0	0	0	0	0	0	3	0	0
							0	0	1	0	1	0	0	0	0	0	0	2
							0	0	0	0	1	0	0	0	0	0	3	0
							0	0	0	3	0	0	0	0	0	0	1	0
							1	0	0	0	1	1	1	0	0	0	0	0
							0	0	0	0	0	0	0	0	0	4	0	0
							0	0	0	0	0	0	0	0	0	0	4	0
							0	0	0	0	0	0	0	0	4	0	0	0
							1	0	2	0	0	0	1	0	0	0	0	0
					Lasiosphaeris	3	0	0	1	0	0	0	1	0	2	0	0	0
							0	0	0	0	0	0	0	0	0	1	3	0
							0	1	0	2	0	0	0	0	0	1	0	0
					Cladorrhinum	7	0	0	0	0	0	0	0	0	0	0	0	4
							0	0	2	0	0	0	0	0	2	0	0	0
							0	0	0	0	0	2	0	0	0	0	2	0
							0	0	0	1	0	0	0	0	0	0	3	0
							0	0	0	0	0	1	0	0	0	0	4	0
							3	0	2	0	0	0	0	0	0	0	0	0
							0	0	0	0	0	1	0	0	0	0	0	4
					Cercophora	4	0	0	0	0	0	0	0	0	0	5	0	0
							0	4	0	0	0	0	0	0	1	0	0	0
							0	0	0	0	0	0	0	0	0	0	5	0
							0	0	0	0	0	0	0	0	0	0	5	0
				Sordariales_fam_Incertae_sedis	*Madurella*	1	0	0	0	0	3	2	0	0	0	0	0	0
			Sordariomycetes_ord_Incertae_sedis	Sordariomycetes_fam_Incertae_sedis	*Pleurotheciella*	1	0	0	0	0	0	0	0	0	0	0	5	0
					Myrmecridium	1	0	0	0	0	0	0	0	0	0	5	0	0
				Annulatascaceae	*Conlarium*	3	0	0	0	5	0	0	0	0	0	0	0	0
							0	0	1	0	2	0	0	0	2	0	0	0
							0	0	0	0	4	0	0	0	0	0	1	0
				Apiosporaceae	*Arthrinium*	5	0	0	0	0	5	0	0	0	0	0	0	0
							0	1	1	0	0	0	0	0	0	1	0	2
							0	0	0	0	0	0	0	0	0	5	0	0
							0	1	0	1	0	0	0	0	0	3	0	0
							0	0	0	1	0	0	0	0	0	0	4	0
				Glomerellaceae	*Colletotrichum*	3	0	0	0	0	1	0	0	0	0	1	0	3
							0	0	0	2	0	1	0	0	0	0	0	2
							0	0	0	0	0	0	5	0	0	0	0	0
				Sordariomycetidae_fam_Incertae_sedis	*Atractospora*	1	0	0	2	0	0	0	0	0	0	2	1	0
			Trichosphaeriales	Trichosphaeriales_fam_Incertae_sedis	*Nigrospora*	2	0	0	0	0	0	0	0	0	0	5	0	0
							0	1	3	0	0	0	0	0	1	0	0	0
			Xylariales	Xylariaceae	*Daldinia*	1	0	0	0	0	0	5	0	0	0	0	0	0
				Xylariales_fam_Incertae_sedis	*Monographella*	4	0	0	5	0	0	0	0	0	0	0	0	0
							0	0	0	2	0	0	0	0	0	0	3	0
							0	0	0	0	0	0	0	0	0	0	2	4
							0	0	0	0	0	0	0	0	0	4	2	0
					Microdochium	4	0	0	0	1	0	1	0	0	0	1	0	3
							0	0	0	0	0	0	0	0	0	0	0	6
							0	0	0	6	0	0	0	0	0	0	0	0
							0	0	0	3	3	0	0	0	0	0	0	0
		Taphrinomycetes	Taphrinales	Protomycetaceae	*Protomyces*	2	0	0	0	0	6	0	0	0	0	0	0	0
							0	0	0	0	0	0	0	0	6	0	0	0
				Taphrinaceae	*Taphrina*	1	0	0	0	1	1	0	0	1	1	1	1	0
				Agaricaceae	*Tulostoma*	1	0	0	0	0	0	0	0	0	1	0	5	0
					Lycoperdon	1	0	0	0	3	0	0	0	0	0	0	3	0
					Echinoderma	1	0	2	0	0	1	0	0	0	0	0	3	0
					Cyathus	1	0	3	0	0	0	0	0	0	3	0	0	0
					Chlorophyllum	1	0	0	3	0	0	0	1	0	2	0	0	0
					Bovista	1	4	2	0	0	0	0	0	0	0	0	0	0
					Agaricus	1	0	0	0	0	0	1	0	0	0	0	3	2
							0	0	0	2	0	0	0	0	0	0	4	0
							0	0	0	0	0	3	0	0	0	0	0	3

		Agaricomycetes	Agaricales	Bolbitiaceae	*Conocybe*	8	0	0	2	2	0	0	0	0	0	0	2	0
							0	0	0	0	0	5	0	0	0	1	0	0
							0	0	0	6	0	0	0	0	0	0	0	0
							0	0	6	0	0	0	0	0	0	0	0	0
							0	0	1	0	0	0	0	0	0	0	0	5
							0	0	0	0	0	7	0	0	0	0	0	0
				Entolomataceae	*Entoloma*	4	0	0	0	0	0	0	0	0	0	0	7	0
							0	0	7	0	0	0	0	0	0	0	0	0
							0	0	0	5	0	0	0	0	0	0	2	0
							0	0	0	0	0	2	0	0	0	1	1	3
				Marasmiaceae	*Marasmius*	2	0	0	7	0	0	0	0	0	0	0	0	0
							0	0	0	0	7	0	0	0	0	0	0	0
				Mycenaceae	*Mycena*	1	0	0	0	0	7	0	0	0	0	0	0	0
				Niaceae	*Flagelloscypha*	2	0	2	4	0	0	0	0	0	1	0	0	0
							0	1	2	1	0	0	0	1	1	0	1	0
				Omphalotaceae	*Omphalotus*	3	0	0	4	0	0	0	0	0	1	1	1	0
							0	0	0	0	1	5	0	0	0	1	0	0
							0	4	2	0	1	0	0	0	1	0	0	0
				Physalacriaceae	*Armillaria*	1	0	0	8	0	0	0	0	0	0	0	0	0
				Pluteaceae	*Pluteus*	1	0	0	0	4	4	0	0	0	0	0	0	0
				Psathyrellaceae	*Psathyrella*	3	8	0	0	0	0	0	0	0	0	0	0	0
							0	0	1	3	0	0	0	0	0	0	2	2
							4	0	2	0	0	0	2	0	0	0	0	0
					Parasola	1	0	0	8	0	0	0	0	0	0	0	0	0
					Coprinopsis	5	0	0	2	0	0	2	0	0	0	4	0	0
							0	6	1	0	0	0	1	0	0	0	0	0
							1	4	2	0	0	0	0	0	1	0	0	0
							0	0	1	3	3	0	0	0	2	0	0	0
							1	1	0	0	0	2	0	4	1	0	0	0
					Coprinellus	5	0	1	6	0	1	0	0	0	1	0	0	0
							0	0	0	0	0	0	0	0	0	0	3	6
							0	1	5	0	0	0	1	0	2	0	0	0
							1	0	1	0	0	1	0	0	0	0	4	2
							0	0	0	0	9	0	0	0	0	0	0	0
				Strophariaceae	*Psilocybe*	1	0	0	0	0	0	0	0	0	0	7	2	0
					Pholiota	2	0	0	0	0	9	0	0	0	0	0	0	0
							0	0	0	0	1	0	0	0	0	0	8	0
					Hypholoma	1	0	0	0	0	9	0	0	0	0	0	0	0
					Hebeloma	1	1	0	0	0	0	0	0	0	0	0	0	8
					Gymnopilus	1	0	0	6	0	0	0	0	0	3	0	0	0
					Agrocybe	1	3	6	1	0	0	0	0	0	0	0	0	0
				Tricholomataceae	*Paralepista*	1	2	5	0	0	0	0	0	0	3	0	0	0
					Lepista	1	0	0	10	0	0	0	0	0	0	0	0	0
			Atheliales	Atheliaceae	*Athelia*	1	0	0	0	0	2	0	0	0	0	0	0	8
					Amphinema	1	0	0	0	0	10	0	0	0	0	0	0	0
			Boletales	Sclerodermataceae	*Scleroderma*	1	0	0	0	0	10	0	0	0	0	0	0	0
					Pisolithus	2	0	0	0	0	0	0	0	0	3	0	2	5
							0	0	0	0	0	3	0	0	0	0	0	7
				Suillaceae	*Suillus*	4	0	0	0	3	0	0	0	0	0	7	0	0
							0	0	0	0	0	0	0	0	5	0	0	5
							0	0	0	1	0	0	0	0	0	0	10	0
							5	0	3	0	0	1	2	0	0	0	0	0
			Cantharellales	Cantharellales_fam_Incertae_sedis	*Minimedusa*	4	1	9	1	0	0	0	0	0	0	0	0	0
							0	1	0	3	0	3	0	0	0	3	1	0
							4	0	0	3	0	0	0	0	0	0	0	4
							2	2	0	0	0	7	0	0	0	0	0	0
				Ceratobasidiaceae	*Thanatephorus*	1	0	0	0	1	0	0	0	0	0	0	10	0
					Rhizoctonia	1	0	0	0	1	0	2	0	0	0	2	0	6
				Hydnaceae	*Hydnum*	1	1	1	7	0	0	0	0	1	2	0	0	0
			Corticiales	Corticiaceae	*Waitea*	1	0	0	0	0	7	0	0	0	0	0	5	0
			Geastrales	Geastraceae	*Sphaerobolus*	1	0	0	1	0	7	0	0	0	0	2	0	2
			Phallales	Phallaceae	*Phallus*	2	0	0	0	10	0	0	0	0	0	2	0	0
							0	0	0	10	0	0	0	0	0	0	0	2
					Clathrus	1	0	0	0	0	0	11	0	0	0	1	0	0
			Polyporales	Ganodermataceae	*Ganoderma*	2	0	0	0	0	0	0	0	11	0	0	1	0
							0	0	3	0	0	0	0	0	6	0	3	0
				Meripilaceae	*Rigidoporus*	1	0	0	1	4	0	0	2	0	0	1	4	0
				Meruliaceae	*Steccherinum*	1	0	0	0	0	0	0	0	0	0	13	0	0
					Abortiporus	1	0	0	0	0	0	0	0	0	0	13	0	0
				Polyporaceae	*Skeletocutis*	1	0	0	0	9	0	2	0	0	0	0	2	0
			Russulales	Peniophoraceae	*Peniophora*	1	0	0	0	0	0	10	0	0	0	3	0	0

	Basidiomycota		Sebacinales	Sebacinaceae	*Efibulobasidium*	1	0	13	0	0	0	0	0	0	0	0	0	0
				Sebacinales_Group_B	*Serendipita*	1	4	0	0	0	0	0	3	0	6	0	0	0
					Piriformospora	1	0	0	0	7	2	4	0	0	0	0	0	0
			Thelephorales	Thelephoraceae	*Tomentella*	1	0	0	0	14	0	0	0	0	0	0	0	0
			Trechisporales	Hydnodontaceae	*Trechispora*	4	3	8	2	0	0	0	0	1	0	0	0	0
							0	0	0	7	0	0	0	0	0	0	7	0
							0	0	0	0	0	0	0	0	0	14	0	0
							0	0	0	0	8	0	0	0	0	0	0	7
					Brevicellicium	1	0	0	15	0	0	0	0	0	0	0	0	0
		Agaricostilbomycetes	Agaricostilbales	Chionosphaeraceae	*Kurtzmanomyces*	1	0	6	0	0	0	0	0	5	2	0	0	2
		Cystobasidiomycetes	Cystobasidiales	Cystobasidiaceae	*Occultifur*	1	0	2	2	3	6	1	0	0	0	0	0	1
					Cystobasidium	2	0	0	0	2	2	0	0	0	0	7	0	4
							0	0	1	1	3	0	1	3	0	0	3	3
			Cystobasidiomycetes_ord_Incertae_sedis	Buckleyzymaceae	*Buckleyzyma*	1	0	0	0	0	5	1	0	0	0	0	7	2
				Cystobasidiomycetes_fam_Incertae_sedis	*Sakaguchia*	2	0	0	0	4	4	4	0	0	0	0	1	3
							0	0	0	0	1	0	0	0	0	0	3	12
			Erythrobasidiales	Erythrobasidiales_fam_Incertae_sedis	*Erythrobasidium*	1	0	0	0	0	0	0	0	0	0	0	16	0
		Exobasidiomycetes	Entylomatales	Entylomataceae	*Entyloma*	1	0	0	0	0	0	0	0	0	0	0	16	0
			Exobasidiomycetidae_ord_Incertae_sedis	Exobasidiomycetidae_fam_Incertae_sedis	*Tilletiopsis*	1	0	0	0	6	3	3	0	0	0	5	0	0
			Georgefischeriales	Gjaerumiaceae	*Gjaerumia*	1	0	0	0	0	17	0	0	0	0	0	0	0
			Microstromatales	Microstromataceae	*Microstroma*	2	0	3	1	0	0	0	5	5	0	0	3	0
							1	1	13	0	0	0	0	0	2	0	0	0
		Microbotryomycetes	Leucosporidiales	Leucosporidiaceae	*Mastigobasidium*	1	0	0	0	17	0	0	0	0	0	0	0	0
			Microbotryales	Microbotryaceae	*Sphacelotheca*	1	0	0	0	0	0	0	0	0	2	0	15	0
			Sporidiobolales	Sporidiobolales_fam_Incertae_sedis	*Sporobolomyces*	4	0	0	1	0	8	1	0	1	0	1	5	1
							1	4	6	0	0	0	0	1	3	0	3	0
							0	0	6	0	0	0	4	0	9	0	0	0
							0	0	0	0	19	0	0	0	0	0	0	0
					Rhodotorula	10	5	1	8	0	0	0	0	3	0	0	1	1
							0	0	0	5	5	0	0	0	0	0	1	8
							0	0	0	0	0	0	0	0	0	0	2	17
							0	0	0	0	9	0	0	0	0	3	8	0
							0	0	0	0	0	0	0	0	0	1	19	0
							1	0	10	0	0	0	2	0	7	0	0	0
							2	15	0	0	0	0	3	0	0	0	1	0
							0	0	0	3	0	3	0	0	0	14	1	0
							3	0	0	0	0	18	0	0	0	0	0	0
							4	0	0	0	0	0	0	0	0	4	5	8
		Pucciniomycetes	Pucciniales	Pucciniaceae	*Puccinia*	2	0	0	0	15	7	0	0	0	0	0	0	0
							0	0	22	0	0	0	0	0	0	0	0	0
		Tremellomycetes	Cystofilobasidiales	Cystofilobasidiaceae	*Udeniomyces*	1	0	0	0	17	6	0	0	0	0	0	0	0
					Phaffia	1	1	2	2	0	0	0	2	0	3	1	12	0
					Cystofilobasidium	3	0	0	0	23	0	0	0	0	0	0	1	0
							0	0	24	0	0	0	0	0	0	0	0	0
							0	0	0	3	3	1	0	0	0	0	3	15
			Filobasidiales	Filobasidiaceae	*Filobasidium*	1	2	5	7	0	0	0	4	7	0	0	0	0
				Piskurozymaceae	*Solicoccozyma*	1	0	0	26	0	0	0	0	0	0	0	0	0
			Tremellales	Tremellales_fam_Incertae_sedis	*Tremella*	1	0	6	9	0	0	0	2	0	4	0	5	0
					Papiliotrema	2	0	0	0	1	0	1	0	0	0	9	8	8
							0	0	0	0	0	0	0	0	0	0	5	22
					Hannaella	3	0	0	2	1	6	6	1	0	0	1	8	2
							0	0	0	25	0	0	0	0	0	0	2	0
							0	0	2	10	1	7	0	0	0	2	5	0
					Dioszegia	1	0	0	0	1	1	0	0	0	0	21	2	3
					Cryptococcus	5	0	0	0	11	0	6	0	0	0	0	0	11
							0	0	0	5	7	3	0	0	0	0	12	1
							0	0	0	7	4	0	0	0	0	11	6	0
							0	0	0	16	1	0	0	0	0	0	5	7
							0	0	0	3	3	7	0	0	0	9	6	2
					Bullera	1	0	0	0	0	0	0	0	0	4	5	12	9
			Trichosporonales	Trichosporonaceae	*Trichosporon*	5	0	0	0	0	31	0	0	0	0	0	0	0
							0	0	0	9	2	0	0	0	0	0	8	13
							0	0	0	0	0	4	0	0	0	0	29	0
							0	1	1	12	11	0	2	1	0	0	7	0
							0	0	12	1	3	0	0	1	15	3	0	0
		Ustilaginomycetes	Urocystidales	Glomosporiaceae	*Glomosporium*	1	0	0	0	34	1	0	0	0	0	1	0	0
			Ustilaginales	Anthracoideaceae	*Farysia*	1	26	4	6	0	0	0	0	0	0	0	0	0
				Ustilaginaceae	*Ustilago*	1	0	4	0	0	0	31	0	0	0	0	0	1
					Sporisorium	2	7	0	16	0	15	0	0	0	0	0	0	0
							0	0	0	2	0	0	0	0	0	0	34	2
					Anthracocystis	2	0	0	7	11	0	0	1	0	0	0	5	14

					Anthracocystis	2	0	0	38	0	0	0	0	0	0	0	0	0
		Ustilaginomycotina_cls_Incertae_sedis	Malasseziales	Malasseziaceae	*Malassezia*	3	0	0	3	0	5	1	0	0	4	11	14	0
							0	0	0	0	0	0	0	0	0	26	0	12
							0	0	0	1	0	0	0	0	0	6	31	0
		Wallemiomycetes	Geminibasidiales	Geminibasidiaceae	*Geminibasidium*	3	0	0	0	0	8	6	0	0	0	0	24	1
							3	0	10	0	0	0	20	8	0	0	0	0
							7	0	1	6	0	1	1	0	0	24	1	0
			Wallemiales	Wallemiaceae	*Wallemia*	4	0	0	0	0	0	34	0	0	0	6	1	0
							0	0	0	0	0	0	0	0	0	0	25	17
							0	0	0	0	5	0	0	0	0	29	5	3
							0	2	7	3	0	0	0	0	0	0	1	30
	Chytridiomycota	Chytridiomycetes	Lobulomycetales	Lobulomycetaceae	*Lobulomyces*	3	1	12	7	3	3	0	3	0	12	1	0	1
							2	5	2	3	1	4	6	0	1	5	3	11
							0	0	2	17	4	1	0	0	2	0	18	0
			Olpidiales	Olpidiaceae	*Olpidium*	4	5	3	6	0	5	3	13	2	7	0	0	0
							3	0	0	3	0	0	0	0	0	30	6	3
							2	4	4	6	4	9	3	1	2	0	10	0
							1	3	1	10	0	10	0	0	0	4	3	13
			Rhizophlyctidales	Rhizophlyctidaceae	*Rhizophlyctis*	11	1	5	19	1	6	2	2	4	2	0	1	2
							0	0	0	0	6	0	0	0	0	22	12	6
							0	0	0	35	5	0	0	0	0	0	6	0
							0	0	8	2	0	0	0	2	0	29	5	0
							0	0	0	3	0	21	0	0	0	8	3	11
							0	0	0	3	8	15	0	0	0	0	11	10
							0	0	0	0	0	0	0	0	0	37	0	11
							0	0	0	45	4	0	0	0	0	0	0	0
							0	0	0	50	0	0	0	0	0	0	0	0
							0	0	0	39	5	0	0	0	0	0	6	0
							0	0	0	0	0	0	0	0	0	0	0	51
				Sonoraphlyctidaceae	*Sonoraphlyctis*	1	0	0	0	0	0	0	0	1	1	44	5	0
			Rhizophydiales	Alphamycetaceae	*Betamyces*	1	0	0	0	28	6	0	0	0	0	0	14	3
					Angulomyces	1	0	0	0	1	8	7	0	0	0	26	9	1
				Rhizophydiaceae	*Rhizophydium*	3	2	39	5	0	0	0	0	0	6	0	0	0
							15	11	0	0	0	0	9	9	5	0	1	3
							0	0	3	2	17	13	1	0	2	0	10	5
				Rhizophydiales_fam_Incertae_sedis	*Operculomyces*	2	4	7	32	0	0	0	0	6	5	0	0	0
							0	4	4	8	6	0	7	4	4	0	8	9
			Spizellomycetales	Spizellomycetaceae	*Spizellomyces*	8	0	0	0	7	0	10	0	0	0	20	14	3
							0	0	12	0	9	2	0	0	21	0	12	0
							0	0	0	0	47	4	0	0	0	0	6	0
							0	0	0	5	4	0	0	0	0	44	5	0
							0	1	1	15	16	7	0	0	0	17	5	0
							0	0	0	11	51	0	0	0	0	0	0	0
							0	0	2	0	0	0	0	52	10	0	0	0
							0	0	0	47	0	1	0	0	0	0	16	0
					Powellomyces	1	0	0	0	29	23	0	0	0	0	5	8	1
					Kochiomyces	1	0	1	0	29	12	12	0	0	2	0	9	2
			Diversisporales	Acaulosporaceae	*Acaulospora*	2	0	0	0	61	6	0	0	0	0	0	0	0
							0	3	0	12	26	4	0	0	0	2	6	15
				Diversisporaceae	*Diversispora*	4	0	0	0	0	64	4	1	0	0	0	0	0
							11	26	9	2	0	0	9	12	0	0	2	0
							0	1	0	9	8	3	0	0	2	9	29	10
							0	0	0	40	17	4	0	0	0	12	0	1
				Gigasporaceae	*Scutellospora*	2	0	0	1	65	3	0	0	0	0	0	1	5
							1	6	2	22	2	11	4	2	3	0	24	1
					Gigaspora	24	9	11	20	0	0	16	0	0	22	0	0	0
							3	1	7	23	22	7	7	0	1	5	3	0
							0	0	0	20	23	23	0	0	0	6	8	1
							1	1	0	72	6	1	0	0	0	1	4	2
							0	0	0	0	31	4	7	0	0	40	0	6
							0	0	0	14	16	18	0	0	0	0	3	39
							5	27	11	2	0	2	18	4	10	2	3	7
							2	1	0	12	0	20	0	0	2	0	20	35
							0	0	0	2	3	21	0	0	2	38	14	14
							0	15	27	0	0	0	33	15	5	0	0	0
							44	5	18	0	0	0	7	0	23	0	0	0
							0	0	0	4	0	76	0	0	0	0	18	3
							0	0	0	0	1	0	0	0	0	38	20	45
							0	6	0	0	0	11	3	0	1	25	21	40
							0	4	0	4	0	6	0	0	0	6	94	0
							0	0	0	114	0	0	0	0	0	0	1	0

	Glomeromycota	Glomeromycetes					0	0	1	4	2	0	0	0	0	3	109	0
							5	9	39	0	0	0	24	34	2	0	8	0
							0	0	0	7	1	15	0	2	0	38	41	23
							0	0	0	66	8	0	0	9	0	7	19	21
							0	0	1	7	4	2	0	0	0	62	54	1
							0	0	0	22	57	9	0	0	0	16	2	30
							0	5	93	4	34	6	0	1	0	0	0	0
							0	0	0	4	0	10	0	0	0	64	41	27
					Dentiscutata	4	0	4	23	0	0	0	0	0	4	0	115	1
							1	1	5	9	14	55	61	4	0	0	5	0
							0	4	76	3	4	1	0	0	23	16	16	13
							7	0	0	73	20	39	0	0	12	0	0	7
					Cetraspora	3	0	0	0	34	13	5	0	0	0	8	82	21
							0	0	0	4	17	71	0	0	0	67	4	2
							0	0	0	0	1	11	0	0	0	60	55	38
			Glomerales	Claroideoglomeraceae	*Claroideoglomus*	5	0	0	0	71	50	43	0	0	0	0	1	1
							0	0	0	5	26	0	0	0	0	6	10	120
							0	0	1	24	76	17	0	0	0	0	50	0
							3	8	3	26	1	3	0	0	1	42	88	1
							0	0	0	124	22	24	0	0	0	0	8	0
				Glomeraceae	*Septoglomus*	2	1	0	1	40	37	13	0	0	5	0	9	73
							0	0	0	11	47	19	0	0	0	72	18	13
					Rhizophagus	7	10	0	109	0	1	10	15	32	16	0	0	0
							7	56	38	0	58	0	2	28	9	0	0	0
							15	24	0	48	15	53	0	2	1	26	9	19
							129	62	0	0	0	0	20	0	1	0	0	0
							0	1	2	3	104	7	35	15	41	0	5	0
							0	0	0	179	21	4	0	0	0	4	4	2
							0	0	0	155	61	0	0	0	0	0	0	0
					Glomus	8	0	0	0	118	99	0	0	0	0	0	0	1
							8	1	4	19	10	115	0	0	0	1	31	38
							0	0	0	30	17	184	0	0	0	2	0	3
							8	23	54	10	121	13	1	3	1	0	0	5
							0	1	0	12	0	5	1	0	0	97	71	52
							79	0	100	0	0	0	24	34	0	0	1	2
							0	0	0	52	65	25	0	0	1	40	29	28
							4	45	170	1	1	1	1	4	20	0	0	0
					Funneliformis	12	1	0	19	10	70	65	6	3	7	10	35	23
							30	17	5	26	3	3	8	5	2	37	75	41
							0	0	0	7	128	15	1	0	0	69	38	1
							0	0	1	15	29	1	0	0	0	58	134	25
							1	0	0	24	8	14	0	0	0	9	128	88
							55	0	1	0	0	0	215	0	28	0	0	0
							25	49	18	2	0	28	32	37	46	10	20	43
							0	0	0	44	52	108	0	0	0	32	34	57
							0	0	0	62	22	1	0	0	0	3	27	213
							19	3	59	31	55	24	100	2	16	0	21	4
							0	0	0	165	35	82	0	0	0	46	0	8
							0	0	0	0	0	0	0	0	0	0	352	0
			Paraglomerales	Paraglomeraceae	*Paraglomus*	9	40	57	145	2	5	4	9	49	6	9	3	23
							0	0	0	4	228	23	0	0	0	18	77	3
							2	11	214	0	0	0	92	8	27	0	0	0
							0	0	0	34	72	3	0	0	0	0	258	0
							1	0	43	73	36	17	9	0	21	74	34	93
							0	0	5	14	8	34	0	0	3	51	225	62
							0	0	0	96	26	2	0	0	0	2	10	267
							0	0	0	2	20	4	0	0	0	252	36	93
							0	0	2	146	22	60	0	1	10	70	38	71
		Kickxellomycotina_cls_Incertae_sedis	Kickxellales	Kickxellaceae	*Ramicandelaber*	1	1	2	0	151	20	56	5	2	1	40	60	89
					Coemansia	1	0	0	0	13	36	98	1	0	4	239	23	29
							6	426	12	0	0	0	0	0	6	6	0	0
							10	6	162	80	20	18	53	58	39	7	3	6
							0	0	13	34	5	17	14	19	10	39	135	183
							3	37	66	1	33	0	21	199	79	6	25	10
							0	4	0	16	137	6	0	0	0	105	151	91
							0	56	10	39	154	44	15	0	1	31	88	80
							0	0	0	487	6	31	0	0	0	4	1	0
							0	0	0	0	4	0	0	0	0	16	516	0
							0	0	0	11	397	0	0	0	0	10	99	21
							1	1	25	157	11	5	0	0	337	12	0	20
							6	10	34	56	136	93	7	29	6	59	61	80

	Zygomycota	Mortierellomycotina_cls_Incertae_sedis	Mortierellales	Mortierellaceae	*Mortierella*	51	20	1	5	73	110	20	0	0	3	97	179	91
							3	3	2	559	6	2	0	0	1	0	13	12
							0	3	0	277	107	91	1	0	0	46	61	17
							0	0	0	5	2	0	0	0	0	123	379	97
							0	0	1	15	44	0	2	0	0	14	531	14
							0	0	22	14	48	4	0	0	17	247	187	87
							7	119	1	24	39	60	56	0	57	70	188	55
							0	0	0	80	39	238	0	0	0	9	14	318
							39	34	72	106	126	134	31	36	17	11	123	20
							17	30	20	71	70	67	47	109	18	138	50	112
							0	0	0	33	19	609	0	0	3	32	35	24
							11	15	1	41	53	134	2	5	0	182	250	89
							1	0	0	15	734	19	0	0	0	16	6	17
							0	0	0	63	529	117	1	0	0	26	58	24
							0	0	0	581	154	8	0	0	0	12	28	71
							0	0	0	10	0	693	0	0	0	154	1	0
							35	1	308	43	190	0	6	0	106	21	34	120
							52	280	23	51	44	63	36	143	14	88	60	51
							79	172	393	0	6	9	58	154	35	0	1	2
							17	117	6	56	69	108	18	58	0	153	265	51
							416	58	21	28	4	10	125	6	40	93	94	31
							52	15	30	95	16	44	137	375	0	92	75	16
							0	5	5	2	7	0	0	1	10	421	495	8
							7	36	3	175	51	392	34	0	26	86	131	56
							671	120	163	0	0	34	47	4	3	0	0	0
							55	31	26	10	0	90	12	2	16	258	199	396
							4	7	8	68	16	47	3	0	75	335	519	75
							1122	7	17	0	0	0	3	7	10	0	0	0
							9	11	5	294	221	114	3	3	1	205	165	149
							55	203	150	67	44	213	62	65	7	171	154	50
							0	2	215	46	12	207	0	0	36	379	166	196
							0	0	0	282	879	85	0	0	0	106	25	80
							0	0	0	350	767	280	0	0	0	36	49	24
							3	0	0	182	669	122	2	0	1	27	476	108
							19	36	21	62	54	84	14	23	15	1245	74	35
							12	113	15	1282	42	25	9	13	13	120	31	44
							18	19	12	268	174	312	6	20	2	265	382	377
							0	1	5	61	64	237	0	0	0	1071	257	227
							646	653	106	15	11	12	208	82	167	16	9	4
							100	1673	0	0	50	9	16	0	47	67	10	0
		Mucoromycotina_cls_Incertae_sedis	Mucorales	Cunninghamellaceae	*Gongronella*	1	17	195	147	175	96	129	95	41	15	304	673	285
					Cunninghamella	3	19	229	925	208	126	20	164	491	63	30	36	14
							9	171	454	396	635	274	65	78	18	70	69	106
							0	0	7	739	236	107	0	0	3	186	595	521
				Lichtheimiaceae	*Lichtheimia*	1	444	143	126	264	102	155	250	227	93	146	291	190
				Mucoraceae	*Mucor*	9	12	3	15	204	671	50	4	32	4	272	999	290
							17	108	72	856	1244	72	22	44	77	84	131	463
							83	96	139	591	690	208	159	110	173	318	552	304
							117	0	0	1617	245	917	345	1	210	1	1	0
							378	270	678	36	266	105	883	76	203	279	206	125
							16	900	769	95	90	99	126	620	677	44	103	108
							116	397	176	423	554	378	89	100	209	340	807	257
							84	168	266	374	471	415	179	150	60	773	632	381
							0	2	0	240	1001	182	4	0	0	196	2102	267
					Actinomucor	2	2	2	2	9	272	13	29	3	196	54	3443	2
							0	0	3	252	249	1174	0	0	0	259	2549	195
				Rhizopodaceae	*Rhizopus*	5	0	0	2	25	167	3637	0	0	0	1	1904	6
							2	0	5769	0	0	0	1	0	2	0	0	0
							0	0	0	4776	484	1	0	0	0	473	0	127
							274	1160	800	146	341	247	648	891	1594	75	125	89
							146	1098	2932	76	413	19	165	253	1681	82	4	63
				Syncephalastraceae	*Circinella*	1	876	1211	1615	421	907	330	553	323	683	238	510	99
				Umbelopsidaceae	*Umbelopsis*	3	414	4075	902	122	88	267	2495	217	450	352	320	389
							706	1672	4447	164	254	110	1556	1047	1326	61	93	116
							969	1168	1947	329	468	334	908	735	1930	386	1310	1238
		Zygomycota_cls_Incertae_sedis	Basidiobolales	Basidiobolaceae	*Basidiobolus*	2	6	891	24	665	85	42	18	7	89	12667	345	293
							8	5	22369	7	682	13	4	5	29	4	19	0

PC_A- ‘Pigarro’ Conventional plant A; PC_B- ‘Pigarro’ Conventional plant B; PC_C- ‘Pigarro’ Conventional plant C; PO_A- ‘Pigarro’ Organic plant A; PO_B- ‘Pigarro’ Organic plant B; PO_C- ‘Pigarro’ Organic plant C ; SC-A-‘SinPre’ Conventional plant A; SC-B-‘SinPre’ Conventional plant B; SC-C-‘SinPre’ Conventional plant C; SO-A-‘SinPre’ Organic plant A; SO-B-‘SinPre’ Organic plant B; SO-C-‘SinPre’ Organic plant C
