# Supplementary material for: Effect of Low-Input Organic and Conventional Farming Systems on Maize Rhizosphere in Two Portuguese Open-Pollinated Varieties (OPV), “Pigarro” (Improved Landrace) and “SinPre” (a Composite Cross Population)
Source: Front Microbiol. 2021 Feb 26;12:636009. doi: 10.3389/fmicb.2021.636009 (PMC7953162; doi:10.3389/fmicb.2021.636009)
Supplement: Supplementary Table 5 — Fungal and bacterial core rhizosphere microbiota associated with maize. [file Table_5.pdf]

Effect of Low Input Organic and Conventional farming systems on maize rhizosphere in two Portuguese OPV, 'Pigarro' (improved landrace) and 'SinPre' (a Composite Cross Population)

Aitana Ares, Joana Costa*, Carolina Joaquim, Duarte Pintado, Daniela Santos, Monika M. Messmer, Pedro Mendes-Moreira

* Correspondence: Corresponding Author: jcosta@uc.pt

Supplementary Table 5. Fungal and bacterial core rhizosphere microbiota associated with maize.

Fungal core microbiota	Bacterial core microbiota
Acremonium	*Candidatus Koribacter*
Acrocalymma	*Candidatus Solibacter*
Actinomucor	*Edaphobacter*
Alternaria	*Actinoallomurus*
Anthracocystis	*Actinocorallia*
Arthrographis	*Actinokineospora*
Aspergillus	*Actinomadura*
Auxarthron	*Actinomycetospora*
Beauveria	*Actinoplanes*
Bullera	*Actinopolymorpha*
Candida	*Aeromicrobium*
Cercophora	*Agromyces*
Chaetomium	*Amycolatopsis*
Chaetosphaeria	*Arthrobacter*
Chloridium	*Catellatospora*
Chrysosporium	*Cellulomonas*
Chytridiomycota	*Cellulosimicrobium*
Cladophialophora	*Conexibacter*
Cladorrhinum	*Cryocola*
Cladosporium	*Curtobacterium*
Claroideoglomus	*Dactylosporangium*
Clonostachys	*Frankia*
Coniochaeta	*Friedmanniella*
Conlarium	*Geodermatophilus*
Coprinopsis	*Georgenia*
Cryptococcus	*Glycomyces*
Cunninghamella	*Gordonia*
Cystofilobasidium	*Iamia*
Diaporthe	*Kibdelosporangium*
Drechslera	*Kribbella*
Exophiala	*Kutzneria*
Funneliformis	*Lentzea*
Fusarium	*Microbacterium*
Fusicolla	*Microbispora*

Ganoderma	*Microlunatus*
Gibberella	*Modestobacter*
Gigaspora	*Mycobacterium*
Glomeromycota	*Nocardia*
Gongronella	*Nocardioides*
Gymnopilus	*Nonomuraea*
Hypholoma	*Phycicoccus*
Lasiosphaeris	*Pilimelia*
Lecanicillium	*Pimelobacter*
Lipomyces	*Pseudonocardia*
Malassezia	*Rhodococcus*
Mastigobasidium	*Saccharopolyspora*
Metacordyceps	*Saccharothrix*
Metarhizium	*Salinibacterium*
Microascus	*Solirubrobacter*
Microdochium	*Sphaerisporangium*
Minimedusa	*Sporichthya*
Monographella	*Streptacidiphilus*
Mortierella	*Streptomyces*
Mucor	*Streptosporangium*
Myrothecium	*Terracoccus*
Ochroconis	*Virgisporangium*
Oidiodendron	*Xylanimicrobium*
Paecilomyces	*Fimbriimonas*
Papiliotrema	*Adhaeribacter*
Parascedosporium	*Candidatus Amoebophilus*
Penicillium	*Chitinophaga*
Phallus	*Chryseobacterium*
Pisolithus	*Dyadobacter*
Podospora	*Flavisolibacter*
Powellomyces	*Flavobacterium*
Puccinia	*Fluviicola*
Purpureocillium	*Hymenobacter*
Pyrenochaeta	*Niastella*
Rhizophlyctis	*Olivibacter*
Rhizopus	*Pedobacter*
Rhodotorula	*Pontibacter*
Saccharomyces	*Rhodocytophaga*
Scolecobasidium	*Segetibacter*
Scopulariopsis	*Sphingobacterium*
Scytalidium	*Sporocytophaga*
Spizellomyces	*Candidatus Protochlamydia*
Sporobolomyces	*Candidatus Rhabdochlamydia*
Sporothrix	*Parachlamydia*
Stachybotrys	*Caldilinea*
Staphylotrichum	*Kouleothrix*
Suillus	*Candidatus Nitrososphaera*
Talaromyces	*Alicyclobacillus*
Trichoderma	*Alkaliphilus*
Umbelopsis	*Ammoniphilus*

Ustilago	*Bacillus*
Veronaeopsis	*Brevibacillus*
Waitea	*Caloramator*
	Clostridium
	Cohnella
	Coprococcus
	Lysinibacillus
	Paenibacillus
	Pelosinus
	Planifilum
	Rummeliibacillus
	Sediminibacterium
	Shimazuella
	Solibacillus
	Sporosarcina
	Streptococcus
	Symbiobacterium
	Tepidimicrobium
	Turicibacter
	Nitrospira
	A17
	Gemmata
	Pirellula
	Planctomyces
	Achromobacter
	Acinetobacter
	Afifella
	Agrobacterium
	Amaricoccus
	Aquicella
	Arthrospira
	Asticcacaulis
	Balneimonas
	Bdellovibrio
	Bosea
	Bradyrhizobium
	Burkholderia
	Caulobacter
	Cellvibrio
	Chelatococcus
	Cupriavidus
	Devosia
	Dokdonella
	Erwinia
	Geobacter
	Hyphomicrobium
	Inquilinus
	Janthinobacterium
	Kaistia
	Kaistobacter

Labrys
Limnohabitans
Luteibacter
Luteimonas
Lysobacter
Mesorhizobium
Methylibium
Methylobacterium
Mycoplana
Myxococcus
Nannocystis
Nitrosovibrio
Novosphingobium
Ochrobactrum
Oxalobacter
Pedomicrobium
Phaeospirillum
Phenylobacterium
Phyllobacterium
Plesiocystis
Pseudomonas
Pseudoxanthomonas
Ramlibacter
Rhizobium
Rhodobacter
Rhodoferax
Rhodoplanes
Roseococcus
Rubellimicrobium
Skermanella
Sorangium
Sphingobium
Sphingomonas
Sphingopyxis
Stenotrophomonas
Steroidobacter
Thermomonas
Variovorax
Deinococcus
Candidatus Xiphinematobacter
Chthoniobacter
DA101
Ellin506
heteroC45_4W
Luteolibacter
Opitutus
OR-59
Pedosphaera
Prosthecobacter
