# Supplementary material for: Effect of Low-Input Organic and Conventional Farming Systems on Maize Rhizosphere in Two Portuguese Open-Pollinated Varieties (OPV), “Pigarro” (Improved Landrace) and “SinPre” (a Composite Cross Population)
Source: Front Microbiol. 2021 Feb 26;12:636009. doi: 10.3389/fmicb.2021.636009 (PMC7953162; doi:10.3389/fmicb.2021.636009)
Supplement: Supplementary Table 6 — Fungal and bacterial genera specific to the rhizosphere microbiota in each population. [file Table_6.pdf]

Supplementary Materia

Effect of Low Input Organic and Conventional farming systems on maize rhizosphere in two Portuguese OPV, 'Pigarro' (improved landrace) and 'SinPre' (a Composite Cross Population)

Aitana Ares, Joana Costa*, Carolina Joaquim, Duarte Pintado, Daniela Santos, Monika M. Messmer, Pedro Mendes-Moreira
* Correspondence: Corresponding Author: jcosta@uc.pt

Supplementary Table 6. Fungal and bacterial genera specific to the rhizosphere microbiota in each population.

Fungal Genera		Bacterial Genera	
**SinPre**	**Pigarro**	**SinPre**	**Pigarro**
Armillaria	*Abortiporus*	*JG37-AG-70*	*Acanthamoeba*
Ascobolus	*Agaricus*	*Azoarcus*	*Aminobacter*
Athelia	*Agrocybe*	*Crenothrix*	*Anaerobacillus*
Barnettozyma	*Amphinema*	*Haliangium*	*Anaerolinea*
Berkleasmium	*Angulomyces*	*Legionella*	*B-42*
Brevicellicium	*Buergenerula*	*Magnetospirillum*	*Chroococcidiopsis*
Calcarisporiella	*Cladoriella*	*Methanosaeta*	*Chthonomonas*
Cercospora	*Clathrus*	*Oscillochloris*	*Citrobacter*
Chalastospora	*Cosmospora*	*Rudanella*	*Corynebacterium*
Chlorophyllum	*Echinoderma*	*Sedimentibacter*	*Coxiella*
Circinella	*Exserohilum*	*Solitalea*	*Dietzia*
Coemansia	*Gliomastix*	*Sporanaerobacter*	*Elizabethkingia*
Cyathus	*Glomosporium*	*Uliginosibacterium*	*Gracilibacillus*
Dactylaria	*Hydnum*		*Halorhodospira*
Efibulobasidium	*Kodamaea*		*Leadbetterella*
Farysia	*Lepista*		*Methanosarcina*
Geomyces	*Microstroma*		*Methylophaga*
Gjaerumia	*Occultifur*		*Natronincola_Anaerovirgula*
Hebeloma	*Paralepista*		*Pelotomaculum*
Kochiomyces	*Pholiota*		*Perlucidibaca*
Kurtzmanomyces	*Pithoascus*		*Pigmentiphaga*
Lichtheimia	*Pleurostoma*		*Rathayibacter*
Lycoperdon	*Polyschema*		*Salinispora*
Madurella	*Pringsheimia*		*Tatlockia*
Omphalotus	*Protomyces*		*Thermobifida*
Paraconiothyrium	*Psathyrella*		*Thermovenabulum*
Peniophora	*Saccharomycopsis*		*Virgibacillus*
Petriella	*Skeletocutis*		
Phaffia	*Sphacelotheca*		
Pleurotheciella	*Sphaerobolus*		
Pluteus	*Steccherinum*		
Ramicandelaber	*Stylonectria*		
Rectifusarium	*Taphrina*		
Rhizoctonia	*Tomentella*		
Rigidoporus	*Torulaspora*		
Scleroderma	*Tulostoma*		
Sclerotinia	*Verrucaria*		
Septoglomus			
Serendipita			
Tilletiopsis			
Trichophyton			
Volutella			
Wickerhamomyces			
