# Supplementary material for: Effect of Low-Input Organic and Conventional Farming Systems on Maize Rhizosphere in Two Portuguese Open-Pollinated Varieties (OPV), “Pigarro” (Improved Landrace) and “SinPre” (a Composite Cross Population)
Source: Front Microbiol. 2021 Feb 26;12:636009. doi: 10.3389/fmicb.2021.636009 (PMC7953162; doi:10.3389/fmicb.2021.636009)
Supplement: Supplementary Table 7 — Fungal and bacterial genera specific to the rhizosphere microbiota of maize in distinct farming system. [file Table_7.pdf]

Supplementary Material

Effect of Low Input Organic and Conventional farming systems on maize rhizosphere in two Portuguese OPV, 'Pigarro' (improved landrace) and 'SinPre' (a Composite Cross Population)

Aitana Ares, Joana Costa*, Carolina Joaquim, Duarte Pintado, Daniela Santos, Monika M. Messmer, Pedro Mendes-Moreira

* Correspondence: Corresponding Author: jcosta@uc.pt

Supplementary Table 7. Genera relative abundance significatively different between 'Pigarro' and 'SinPre' populations ($P < 0.05$).

Genera	Difference	
	Pigarro	SinPre
Abortiporus - Alternaria	-1233,5	-1406,67
Abortiporus - Aspergillus	-1199,83	-2969,67
Abortiporus - Fusarium	-2897,83	-1764,33
Abortiporus - Mortierella	-2368,33	-1928,67
Abortiporus - Oidiodendron	-4830,5	-
Abortiporus - Penicillium	-1458,67	-893,667
Abortiporus - Podospora	-1033,67	-780,5
Abortiporus - Rhizophlyctis	-1673,83	-1569,83
Abortiporus - Rhizopus	-3952,67	-
Abortiporus - Dentiscutata	-	-546,833
Abortiporus - Exophiala	-	-723,5
Abortiporus - Microdochium	-	-703,5
Abortiporus - Olpidium	-	-667,5
Abortiporus - Cunninghamella	-	-679,5
Acaulospora - Alternaria	-1232,83	-1406,67
Acaulospora - Aspergillus	-1199,17	-2969,67
Acaulospora - Fusarium	-2897,17	-1764,33
Acaulospora - Mortierella	-2367,67	-1928,67
Acaulospora - Oidiodendron	-4829,83	-
Acaulospora - Penicillium	-1458	-893,667
Acaulospora - Podospora	-1033	-780,5
Acaulospora - Rhizophlyctis	-1673,17	-1569,83
Acaulospora - Rhizopus	-3952	-
Acaulospora - Cunninghamella	-	-679,5
Acaulospora - Dentiscutata	-	-546,833
Acaulospora - Exophiala	-	-723,5
Acaulospora - Microdochium	-	-703,5
Acaulospora - Olpidium	-	-667,5
Acremonium - Fusarium	-2669,67	-1512,5
Acremonium - Penicillium	-1230,5	-641,833
Acremonium - Rhizophlyctis	-1445,67	-1318
Acremonium - Rhizopus	-3724,5	-
Acremonium - Alternaria	-	-1154,83
Acremonium - Aspergillus	-	-2717,83
Acremonium - Mortierella	-	-1676,83
Acrocalymma - Alternaria	-1124,5	-
Acrocalymma - Aspergillus	-1090,83	-2882,83
Acrocalymma - Fusarium	-2788,83	-1677,5
Acrocalymma - Mortierella	-2259,33	-1841,83
Acrocalymma - Oidiodendron	-4721,5	-
Acrocalymma - Penicillium	-1349,67	-806,833
Acrocalymma - Rhizophlyctis	-1564,83	-1483
Acrocalymma - Rhizopus	-3843,67	-1319,83
Acrocalymma - Cunninghamella	-	-592,667
Acrocalymma - Exophiala	-	-636,667
Acrocalymma - Microdochium	-	-616,667
Acrocalymma - Olpidium	-	-580,667
Acrocalymma - Podospora	-	-693,667
