# Supplementary material for: Effect of Low-Input Organic and Conventional Farming Systems on Maize Rhizosphere in Two Portuguese Open-Pollinated Varieties (OPV), “Pigarro” (Improved Landrace) and “SinPre” (a Composite Cross Population)
Source: Front Microbiol. 2021 Feb 26;12:636009. doi: 10.3389/fmicb.2021.636009 (PMC7953162; doi:10.3389/fmicb.2021.636009)
Supplement: Supplementary Table 8 — Genera relative abundance significantly different between “Pigarro” and “SinPre” populations (P < 0.05). [file Table_8.pdf]

Supplementary Material

Effect of Low Input Organic and Conventional farming systems on maize rhizosphere in two Portuguese OPV, 'Pigarro' (improved landrace) and 'SinPre' (a Composite Cross Population)

Aitana Ares, Joana Costa*, Carolina Joaquim, Duarte Pintado, Daniela Santos, Monika M. Messmer, Pedro Mendes-Moreira

* Correspondence: Corresponding Author: jcosta@uc.pt

Supplementary Table 8. Fungal and bacterial genera specific to the rhizosphere microbiota of maize in distinct farming system.

Fungal Genera		Bacterial Genera	
**Conventional**	**Organic**	**Conventional**	**Organic**
Agaricus	*Abortiporus*	*Actinotalea*	*Acanthamoeba*
Armillaria	*Acaulospora*	*Azoarcus*	*Aminobacter*
Ascobolus	*Acrostalagmus*	*B-42*	*Anaerobacillus*
Barnettozyma	*Agrocybe*	*Brevundimonas*	*Anaerovorax*
Basidiobolus	*Amphinema*	*Candidatus Entotheonella*	*Anoxybacillus*
Brachyphoris	*Angulomyces*	*Chloronema*	*Blastochloris*
Brevicellicium	*Arachnomyces*	*Citrobacter*	*Chroococcidiopsis*
Chalastospora	*Articulospora*	*Coxiella*	*Corynebacterium*
Chlorophyllum	*Athelia*	*Dermacoccus*	*Crenothrix*
Cosmospora	*Betamyces*	*Exiguobacterium*	*Dechloromonas*
Daldinia	*Bovista*	*Haliangium*	*Demequina*
Lectera	*Buckleyzyma*	*Halorhodospira*	*Desulfosporosinus*
Lepista	*Buergenerula*	*Kineococcus*	*Desulfotomaculum*
Paraconiothyrium	*Calcarisporiella*	*Nitrosococcus*	*Dietzia*
Paralepista	*Cercospora*	*Oscillochloris*	*Emticicia*
Peniophora	*Cetraspora*	*Paenisporosarcina*	*Flavihumibacter*
Piriformospora	*Circinella*	*Parapedobacter*	*Gracilibacillus*
Pluteus	*Cladoriella*	*Parvibaculum*	*Gracilibacter*
Rectifusarium	*Clathrus*	*Ralstonia*	*JG37-AG-70*
Rigidoporus	*Coemansia*	*Rathayibacter*	*Larkinella*
Septoglomus	*Conocybe*	*Rickettsia*	*Leadbetterella*
Sonoraphlyctis	*Cryptococcus*	*Rubricoccus*	*Lutispora*
Sphaerobolus	*Cyathus*	*Sinomonas*	*Magnetospirillum*
Thielavia	*Cyphellophora*	*Sporomusa*	*Methanosaeta*
Volutella	*Dactylaria*	*Tatlockia*	*Methanosarcina*
	Dactylonectria	*Tsukamurella*	*Methylophaga*
	Debaryomyces	*Uliginosibacterium*	*Mycetocola*
	Dentiscutata	*Yonghaparkia*	*Natronincola_Anaerovirgula*
	Diversispora		*Nodularia*
	Echinoderma		*Oceanobacillus*
	Efibulobasidium		*Perlucidibaca*
	Entyloma		*Pigmentiphaga*
	Erythrobasidium		*Planomicrobium*
	Exserohilum		*Rubrobacter*

	Filobasidium		*Rudanella*
	Flagelloscypha		*Ruminococcus*
	Geminibasidium		*Saccharomonospora*
	Geomyces		*Salinispora*
	Gjaerumia		*Sedimentibacter*
	Gliomastix		*SMB53*
	Glomosporium		*Solitalea*
	Glomus		*Spirochaeta*
	Hanseniaspora		*Spirosoma*
	Hebeloma		*Sporanaerobacter*
	Helicoma		*Sporotomaculum*
	Hydnum		*Thermobifida*
	Kochiomyces		*Thermobispora*
	Kodamaea		*Thermovenabulum*
	Kurtzmanomyces		*Thiobacillus*
	Leptodiscella		*Turneriella*
	Lichtheimia		*Virgibacillus*
	Lophiostoma		*WCHB1-84*
	Lycoperdon		
	Macrophomina		
	Marasmius		
	Mariannaea		
	Microstroma		
	Mycena		
	Myrmecridium		
	Neobulgaria		
	Nigrospora		
	Occultifur		
	Omphalotus		
	Operculomyces		
	Ophiosphaerella		
	Paraglomus		
	Parasola		
	Periconia		
	Petriella		
	Phaffia		
	Phialocephala		
	Pholiota		
	Pithoascus		
	Pleurostoma		
	Pleurotheciella		
	Polyschema		
	Pringsheimia		
	Protomyces		
	Psathyrella		
	Pseudopyricularia		
	Psilocybe		
	Ramicandelaber		
	Ramularia		
	Rhizoctonia		

	Saccharicola		
	Saccharomycopsis		
	Sarocladium		
	Schizothecium		
	Scleroderma		
	Sclerotinia		
	Scutellospora		
	Selinia		
	Setophaeosphaeria		
	Skeletocutis		
	Sphacelotheca		
	Spiromastix		
	Sporisorium		
	Steccherinum		
	Stylonectria		
	Taphrina		
	Tetracladium		
	Tetraplosphaeria		
	Tilletiopsis		
	Tomentella		
	Torulaspora		
	Tremella		
	Trichophyton		
	Tulostoma		
	Verrucaria		
	Wickerhamomyces		
